# High-resolution AP-SMALDI MSI as a tool for drug imaging in *Schistosoma mansoni*

**DOI:** 10.1007/s00216-021-03230-w

**Published:** 2021-03-15

**Authors:** Annika S. Mokosch, Stefanie Gerbig, Christoph G. Grevelding, Simone Haeberlein, Bernhard Spengler

**Affiliations:** 1 Institute of Inorganic and Analytical Chemistry, Justus Liebig University Giessen, 35392 Giessen, Germany; 2 Institute of Parasitology, BFS, Justus Liebig University Giessen, 35392 Giessen, Germany

**Keywords:** *Schistosoma mansoni*, MALDI mass spectrometry imaging, Drug imaging, Drug repurposing, Neglected tropical diseases, Imatinib

## Abstract

*Schistosoma mansoni* is a parasitic flatworm causing schistosomiasis, an infectious disease affecting several hundred million people worldwide. Schistosomes live dioeciously, and upon pairing with the male, the female starts massive egg production, which causes pathology. Praziquantel (PZQ) is the only drug used, but it has an inherent risk of resistance development. Therefore, alternatives are needed. In the context of drug repurposing, the cancer drug imatinib was tested, showing high efficacy against *S. mansoni* in vitro. Besides the gonads, imatinib mainly affected the integrity of the intestine in males and females. In this study, we investigated the potential uptake and distribution of imatinib in adult schistosomes including its distribution kinetics. To this end, we applied for the first time atmospheric-pressure scanning microprobe matrix-assisted laser desorption/ionization mass spectrometry imaging (AP-SMALDI MSI) for drug imaging in paired *S. mansoni*. Our results indicate that imatinib was present in the esophagus and intestine of the male as early as 20 min after in vitro exposure, suggesting an oral uptake route. After one hour, the drug was also found inside the paired female. The detection of the main metabolite, N-desmethyl imatinib, indicated metabolization of the drug. Additionally, a marker signal for the female ovary was successfully applied to facilitate further conclusions regarding organ tropism of imatinib. Our results demonstrate that AP-SMALDI MSI is a useful method to study the uptake, tissue distribution, and metabolization of imatinib in *S. mansoni*. The results suggest using AP-SMALDI MSI also for investigating other antiparasitic compounds and their metabolites in schistosomes and other parasites.

**Supplementary Information:**

The online version contains supplementary material available at 10.1007/s00216-021-03230-w.

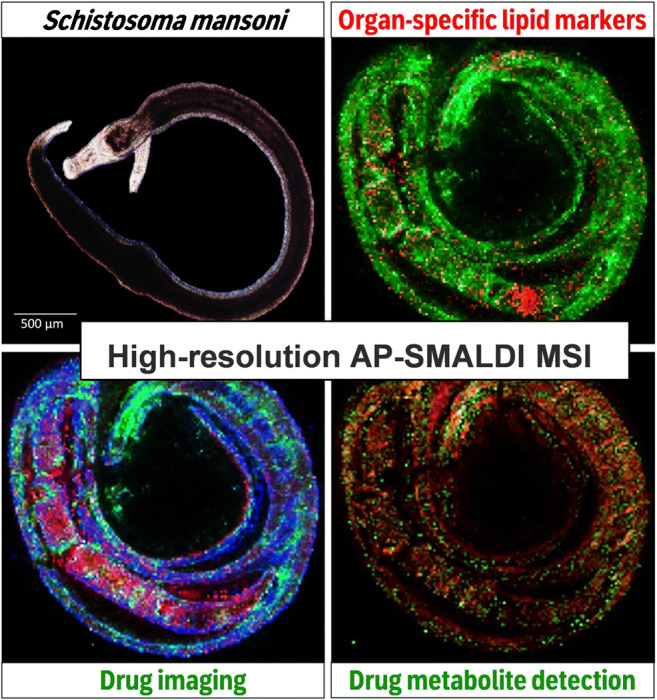

## Introduction

Schistosomiasis is a disease caused by trematodes of the genus *Schistosoma*, with more than 200 million people affected and around 200,000 annual deaths globally. The WHO has listed schistosomiasis as one of the neglected tropical diseases (NTDs) [1–4]. Interestingly, schistosomes have evolved two sexes, in contrast to almost all other parasitic flatworms which are hermaphrodites [5]. For pairing, the female becomes embraced by the male’s body and locates in the ventral groove of the male, the gynaecophoric canal. A constant pairing contact of male and female worms is required to induce and maintain the sexual maturation of the female [5]. Following pairing, the female produces hundreds of eggs per day, which are released into the bloodstream of the final host, such as humans. Some of the eggs reach the gut lumen and are released into the environment to continue the life cycle of the parasite. The rest of the eggs, however, migrate via the bloodstream and get trapped in different organs such as the spleen and liver. Here, these eggs cause granuloma formation and inflammatory processes, finally leading to liver fibrosis [6]. For treatment of schistosomiasis, praziquantel (PZQ) is used as the only available drug effective against all schistosome species relevant to humans [7]. However, PZQ does not prevent reinfection, and indications of resistance development against this drug have been recorded [8, 9]. To date, there is no vaccine available, which alarmingly limits disease control [10, 11].

Several new drugs against *S. mansoni* are currently under investigation, including newly developed substances and repurposed drugs. One of these studied drugs is imatinib, a protein tyrosine-kinase (PTK) inhibitor targeting Abl-family PTKs [12, 13]. Imatinib, also known as Gleevec or Glivec (Novartis, Basel, Switzerland; formerly referred to as STI571 or CGP57148B), is used for therapy of chronic myeloid leukemia and malignant gastrointestinal stroma tumors in humans [12, 14]. In in vitro assays, imatinib has shown additional efficacy against multicellular and unicellular parasites with high relevance to human health such as *S. mansoni* and *S. japonicum* [15, 16] but also against *Echinococcus multilocularis* [17], filaria [18, 19], *Plasmodium* [20, 21], and *Leishmania* [22]. Two Abl orthologs and an Abl/Src hybrid kinase occur in *S. mansoni* and are targets of imatinib [15, 23, 24]. Incubation of *S. mansoni* with concentrations between 10 and 100 μmol/L imatinib resulted in phenotypic changes including bulges and swellings along the entire worm body and a reduction of pairing stability and viability of *S. mansoni* couples as well as degenerations of the gonads and the gastrodermis in both genders [15]. Although the phenotypic effects following imatinib treatment of worms have been analyzed in detail, among the open questions are the following: (i) how is imatinib taken up by the parasite (orally or via a different route), (ii) whether the drug uptake kinetics differ between males and females, (iii) in which tissues does the drug occur, (iv) how does drug tropism correlate with the observed phenotypes, and (v) how is imatinib metabolized in the worm? Providing answers to these questions was central to our study.

Typically applied methods to investigate drug distribution require labeling of the compound, either by fluorescent probes or by radioactive substances [25]. Both techniques are quite expensive and laborious, and attachment of probes to a bioactive compound might influence its chemical behavior. Another possibility is to visualize drug distributions by using mass spectrometry imaging (MSI) [26]. The advantage of mass spectrometric detection is that molecules can mostly be analyzed in their native state without additional labeling. Furthermore, hundreds of other endogenous compounds or drug metabolites can be detected in parallel. Sample preparation includes the sectioning of tissue, which can be complicated for small organisms. Our lab has previously established and optimized this procedure for *S. mansoni* [27]. MSI can be carried out using several ionization techniques, the most widespread being matrix-assisted laser desorption/ionization (MALDI). After coating the sample with a thin layer of a dedicated organic matrix, the pulsed laser beam ablates sample material from the tissue surface in a rasterized fashion [28]. The resulting mass spectrum and the location of the ablated spot on the sample are recorded, allowing the generation of images that show the distributions of compounds throughout the rasterized sample area. Typically, MALDI is optimally suited for the detection of phospholipids and smaller metabolites, but it can also be used for peptides and numerous exogenous molecules [29]. Parallel detection of several hundred endogenous molecules requires highly resolved and accurate detection of *m*/*z* values by the mass spectrometric analyzer to enable the discrimination and assignment of compounds with similar mass [30]. Among the available systems, high-speed time-of-flight mass analyzers are still predominant, but Fourier-transform mass spectrometers are becoming increasingly important for MALDI imaging. Orbital trapping and ion cyclotron resonance (ICR) are techniques that provide the highest mass accuracy and resolution. While ICR is superior to Orbitraps concerning the aforementioned parameters, they require relatively costly maintenance, and measurement speed at maximum resolution is low. Therefore, Orbitrap mass analyzers are becoming more popular for high-mass-resolution analysis of biomolecules in tissue samples [31] at competitive speed [32]. Their superior resolution coupled with speed of analysis makes them very suitable for drug imaging by providing unambiguous identification through accurate mass and fragmentation.

In the present study, we have investigated imatinib distribution in cryosections of *S. mansoni* after refining the sample preparation protocol and applying high-resolution atmospheric-pressure scanning microprobe MALDI MSI (AP-SMALDI MSI). We have analyzed two concentrations of imatinib and several time points after treatment of the worms with the drug. We were able to follow the uptake and distribution of imatinib and its major metabolite in the worm and to address several characteristic anatomical features.

## Materials and methods

### Statement of human and animal rights

Animal experiments using Syrian hamsters (*Mesocricetus auratus*) as model hosts were performed in accordance with the European Convention for the Protection of Vertebrate Animals used for experimental and other scientific purposes (ETS No 123; revised Appendix A). Experiments have been approved by the Regional Council (Regierungspraesidium) Giessen (V54-19 c 20/15 h 02 GI 18/10 Nr. A 14/2017).

### Harvesting of *Schistosoma mansoni*

A Liberian strain of *S. mansoni* was maintained in Syrian hamsters as final host and freshwater snails of the genus *Biomphalaria glabrata* as intermediate host [6, 33]. Eight-week-old hamsters were obtained from Janvier (France) and infected by the “paddling method” [34]. Adult worms were collected at 46 days p.i. by hepatoportal perfusion and cultured in M199 medium (Sigma-Aldrich, Schnelldorf, Germany; supplemented with 10% newborn calf serum (NCS), 1% HEPES [1 M], and 1% ABAM solution [10,000 units penicillin, 10 mg streptomycin, and 25 mg amphotericin B per mL]) at 37 °C and 5% CO_2_.

### In vitro culture experiments

For in vitro culture with imatinib, adult *S. mansoni* were cultured in 6-well plates with 10 worm couples per well in supplemented M199 medium. Imatinib (imatinib mesylate, purity ≥ 98% (HPLC); Enzo Life Sciences, Lorrach, Germany) was dissolved in dH_2_O as 50 mmol/L stock and dissolved in medium to final concentrations of 10–100 μmol/L as indicated in the text. The worms were incubated with imatinib at 37 °C and 5% CO_2_ for up to 48 h, in which the medium and imatinib were refreshed every 24 h. For AP-SMALDI MSI, worms were used within the first 24 h of treatment, for morphological analysis within 48 h. Worm motility and the frequency of separation of worm couples were recorded at the indicated time points using bright-field microscopy (Labovert FS, Leitz, and SC30 camera, Olympus). Worm motility was quantified using a scoring system, following recommendations by WHO-TDR [35], with the scores 3 (normal motility), 2 (reduced motility), 1 (minimal and sporadic movements), and 0 (no movements within 30 s was considered dead).

### Fixation of *Schistosoma mansoni* for AP-SMALDI MSI

After in vitro culture with imatinib for different time periods (see below), worm couples were transferred to plain culture medium using featherweight forceps and washed by a short incubation to remove excess drug and medium. Afterwards, all couples from one well were fixed in 50 μL of a 6.6% solution of glutaraldehyde (grade I, 25% in H_2_O; Sigma-Aldrich) in PBS (≥ 99%, p.a.; Carl Roth, Karlsruhe, Germany) on a glass slide, frozen in liquid nitrogen, and stored at − 80 °C. Two series of measurements with 100 μmol/L (high-concentration treatment group) and 20 μmol/L (low-concentration treatment group) concentrations of imatinib were carried out. The investigated time points were 0 min (control, no imatinib added), 5 min, 20 min, 1 h, 4 h, 12 h, and 24 h for 100 μmol/L and 20 min, 1 h, 4 h, 12 h, and 24 h for 20 μmol/L.

### Sectioning

**Fig. 1:**
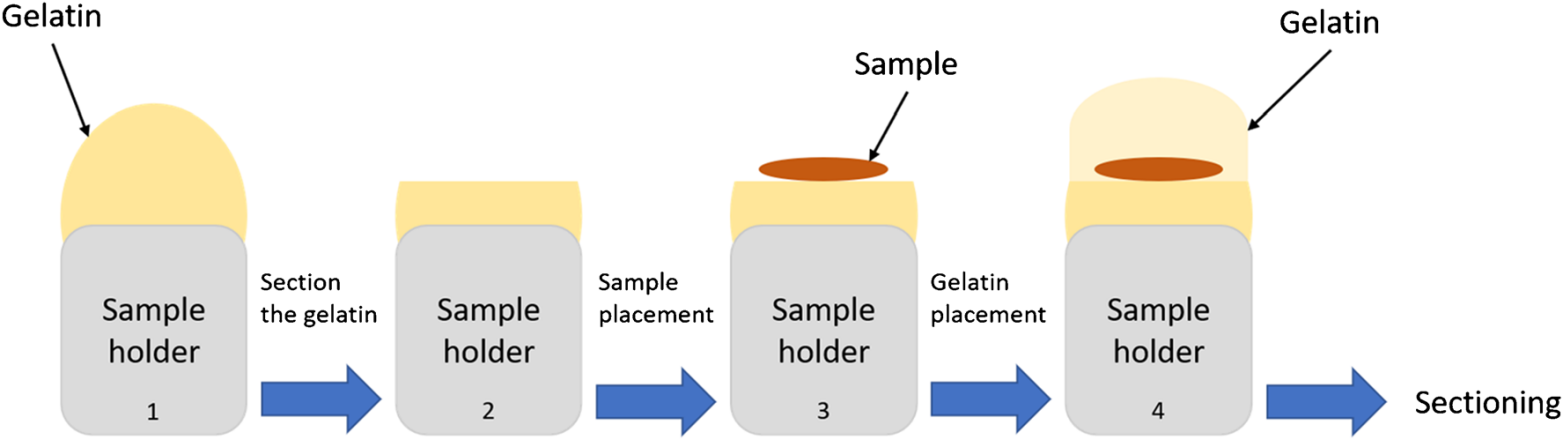
Scheme of sectioning procedure

Sectioning of worms was carried out on a cryostat HM525 (Thermo Fisher Scientific Inc., Waltham, USA). The sectioning protocol was adapted from Kadesch et al. [27]; the adapted protocol is pictured in Fig. 1. Aqueous gelatin solution with a mass concentration of *β* = 80 g/L (water: LC-MS grade, VWR International GmbH, Darmstadt, Germany; gelatin: Pharm. Eur., VWR, Radnor, USA) was prepared. Fifteen microliters of gelatin solution was placed on a sample holder (stainless steel, *d* = 6 mm) and frozen at − 25 °C in the cryotome for 30 min (step 1). Afterwards, the upper part of the droplet was sectioned to form a flat surface (step 2). The fixed and frozen worm couples were thawed in a desiccator at room temperature for 30 min before they were transferred with featherweight forceps in 200 μL water for 30 s to rinse off the residues of the fixative. Subsequently, they were placed on the gelatin plateau and again mounted in the cryotome (step 3). Fifteen microliters of gelatin solution was put on top of the worm couples, then frozen at − 25 °C in the cryotome for 30 min (step 4). After freezing, the samples were cut into sections with a thickness of 40 μm. The quality of the sections was assessed using a digital light microscope (VHX 5000, Keyence, Osaka, Japan), and optical images were recorded. Sections were stored at − 80 °C until further processing.

### AP-SMALDI MSI measurements

Sections were thawed in a desiccator at room temperature for 30 min. They were sprayed with a matrix solution consisting of 2,5-dihydroxybenzoic acid (DHB, Sigma-Aldrich GmbH, St. Louis, USA) in a concentration of *β* (DHB) = 30 mg/mL. The solution was prepared by dissolving DHB in 1:1 (v/v) acetone:water, followed by addition of pure trifluoroacetic acid to obtain a 0.1% acidic solution (acetone, LiChrosolv, Merck, Darmstadt, Germany; TFA, spectroscopy grade, AppliChem GmbH Darmstadt, Deutschland). A SMALDIPrep sprayer (TransMIT GmbH, Giessen, Germany) was used to apply 140 μL of matrix solution to each sample with a flow rate of 10 μL/min at a nitrogen pressure of 1 bar. For the first eleven samples from the first measurement series, 80 μL matrix solution was used. The amount was then increased to 140 μL, which prevented measurement artifacts.

Imaging experiments were performed on a QExactive HF orbital trapping mass spectrometer (Thermo Fisher Scientific, Bremen, Germany) equipped with an autofocusing AP-SMALDI5 AF ion source [36, 37] (TransMIT GmbH, Giessen, Germany). Fifty UV-laser pulses per pixel at a frequency of 100 Hz were used to desorb/ionize the samples. Pixel sizes between 5 and 9 μm were set. Pixel sizes were chosen according to the available measurement time and sample size. Pixelwise autofocusing was used for all measurements. The *m*/*z* range was 250 to 1000 u. All measurements were performed in positive-ion mode with a mass resolution of 240,000 at *m*/*z* 200. A lock mass at *m*/*z* 716.12451, corresponding to [5DHB – 4H_2_O + NH_4_]^+^ was chosen for internal calibration. The ion injection time was set to 500 ms, the s-lens level was set to 100 arbitrary units, and the capillary temperature was chosen to be 250 °C. An acceleration voltage of 3.0 kV was set.

### Data analysis

Q Exactive Tune (version 2.4, Thermo Fisher Scientific, Bremen, Germany) was used to record spectra at the Q Exactive HF mass spectrometer. The software “SMALDI Control” (V1.1–118, TransMIT GmbH, Giessen, Germany) was used to control the stage for image acquisition and for control of the autofocus. XCalibur (Thermo Fisher Scientific, Bremen, Germany) was utilized to display mass spectra. Mirion software package was used for data visualization [38]. The absolute mass variance of spectra was set to 0.005 u, and the bin width of the histogram was set to 0.004 u. Each *m*/*z* signal was normalized to the total ion current (TIC) of the corresponding pixel for image generation. Lipid assignment was carried out using lipid maps [39] and metaspace (https://metaspace2020.eu) [40]. Chemical structures were drawn using ACD/ChemSketch (Advanced Chemistry Development Inc., Toronto, Canada).

### Confocal laser scanning microscopy

Morphologic effects on organs such as intestine and gonads were assessed in detail after 4 h, 24 h, and 48 h of treatment with 100 μmol/L imatinib. To this end, worms were fixed with AFA (66.5% ethanol, 1.1% paraformaldehyde, 2% glacial acetic acid) and stained with carmine red (CertistainH; Merck, Germany) as described before [15, 41]. Stained worms were examined on an inverse confocal laser scanning microscope (CLSM) (Leica TSC SP5; Leica Microsystems, Wetzlar, Germany). Carmine red was excited with an argon-ion laser at 488 nm. Laser power as well as gain and offset of the photomultiplier tube (PMT) was optimized for minimizing possible bleaching effects and for full range intensity coding using the CLUT function (color look-up table) of the Leica LAS AF software. Optical section thickness and background signals were defined by setting the pinhole size to airy unit 1.

## Results and discussion

### Optimal in vitro culture conditions of worms with imatinib for AP-SMALDI MSI studies

Imatinib is known to induce bulges and swellings along the worm body and to destabilize tissue integrity within the gonads and the gastrodermis of *S. mansoni* after incubation times of 24–96 h at 10–100 μmol/L [15]. Using 100 μmol/L, all worm couples separated into individual males and females within 24 h. Exposure times shorter than 24 h have not yet been studied. Because single females are extremely thin, longitudinal sectioning required for AP-SMALDI MSI is difficult to achieve [27]. Therefore, we established earlier time points of treatment that allow both imaging of intact couples by AP-SMALDI MSI and analysis of drug-induced effects on tissue morphology.

**Fig. 2:**
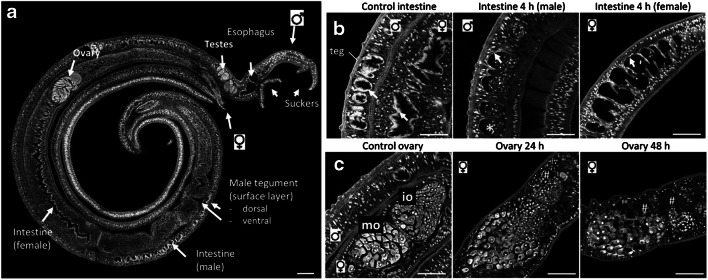
Morphology of untreated and imatinib-treated *S. mansoni*. **a** Untreated control couple of adult *S. mansoni* worms with an overview of the major organs. As typical for schistosome couples, the male surrounded the female. **b** Imatinib (100 μmol/L) destroyed the gastrodermis (arrows) of male and female worms within 4 h treatment. Accumulation of cellular debris (*) was found in the intestinal lumen. **c** Imatinib (100 μmol/L) induced degradation of the female ovary as early as 24 h, and more severely after 48 h treatment (holes, #). io, immature oocytes; mo, mature oocytes; teg, tegument; scale bar, 100 μm (**a**) or 50 μm (**b**, **c**)

Figure 2a provides an overview of the morphology of untreated paired male and female *S. mansoni*, which were of interest in our AP-SMALDI MSI study. Clearly visible are two suckers of the male, which anchor the worm couple in place within the blood vessel and allow for directed movement; the intestinal tract, consisting of the opening in the oral sucker, the connecting esophagus, and the intestine—the latter is covered by a bioactive layer, the gastrodermis, which serves for nutrient uptake; the bioactive outer surface layer, the tegument, which represents the host-parasite interface protecting the parasite from immune attack of the host, and which mediates nutrient uptake; the ventral side of the male tegument is in direct contact with the female; and finally, the gonads (female ovary and male testis), which are essential for reproduction.

To determine the onset of morphological effects on tissues, we incubated *S. mansoni* couples with 100 μmol/L imatinib to establish the optimal duration of treatment for subsequent AP-SMALDI MSI experiments. An increasing impact on worm vitality was found over time: at 1 h, worms lost fitness and detached with their suckers from the ground; at 4 h, the majority of worm couples separated into single male and female worms; from 12 h onward, worm motility started to decrease (see Supplementary Information (ESM) Fig. S1 a-c). In ESM Fig. S1 d, bright-field microscopy images of representative worm couples are depicted. The couples of the control group were paired and attached to the culture well, while after 4 h, the couples were separated, and the males curled up in comparison to the control group. After 24 h of treatment, the couples were still separated, but the males were no longer curled up. By CLSM analysis of carmine-red-stained worms, we found a degradation of the gastrodermis in both male and female worms, as early as 4 h after treatment. This included an accumulation of cellular debris especially in the male intestinal lumen (Fig. 2b) and corresponded to earlier findings at 24 h [11]. This applied also to the disintegration of the gonad tissue, which was visible after a longer exposure of 24 h, and even more prominent at 48 h (Fig. 2c). Based on these findings, we focused on time points up to 12 h for the subsequent AP-SMALDI MSI studies.

### Improved cryosectioning for worm samples

The availability of adequate sections is very important for MSI applications. Since *S. mansoni* worms are very small (< 10 mm in length; 200–500 μm in diameter) and soft, sectioning is challenging. Kadesch et al. [27] developed a protocol for worm couples and individual worms, based on gelatine embedding and cryosectioning. Treatment with imatinib, however, drastically changed and destabilized the structure of treated worms (Fig. 2, ESM Fig. S1). This caused additional difficulties during sectioning and made adaptions of the sectioning protocol necessary. In the original procedure, the schistosomes were placed directly on a metal sample holder, while we used an underlying gelatin plateau in our approach (Fig. 1).

**Fig. 3:**
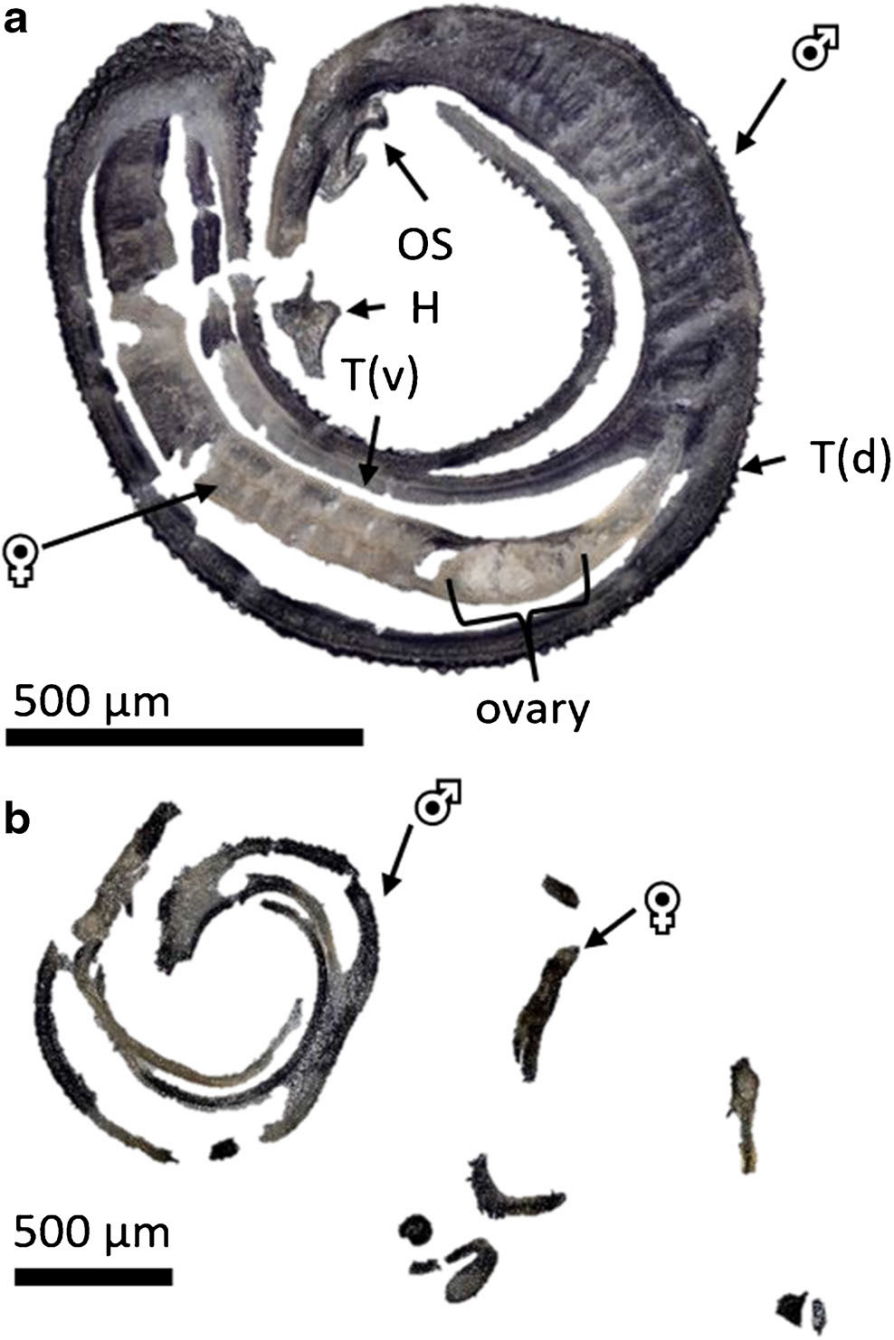
**a** Optical image of a cryosection of a representative *S. mansoni* couple (100 μmol/L imatinib, 1 h incubation time). VS = ventral sucker, OS = oral sucker, T(v) = tegument ventral (in contact with the female body), and T(d) = tegument dorsal (host-parasite interface). **b** Optical image of a cryosection of a separated *S. mansoni* couple (100 μmol/L imatinib, 12 h incubation time)

In Fig. 3a, a section obtained with the adapted method is shown. The sectioned couple was treated with a 100-μmol/L imatinib solution for 1 h. Male and female worms can still be distinguished, and it is also possible to identify the head and the oral suckers of the male, indicating the orientation of the worms within the section. Within the female, the ovary is clearly visible as a lighter, delimited anatomic structure. There are some cracks in the cryosections due to the fragile nature of the imatinib-treated worms. This effect became more pronounced with increasing drug concentrations and incubation periods, leading to the separation of the male and female worms (Fig. 3b). Optical images of all prepared cryosections at different imatinib concentrations and time points are presented as ESM (Figs. S2, S4, and S5).

Using an imatinib concentration of 100 μmol/L, worm couples separated after an incubation time of 4 h. Since separated worms were more difficult to section, the imatinib concentration for the second measurement series was lowered to 20 μmol/L. This reduced the frequency of separation of couples within the first 24 h of treatment. In a standard dilution series, imatinib was detected up to a concentration of 0.2 μmol/L (ESM Fig. S6).

In conclusion, the adapted sectioning protocol resulted in reproducible and authentic sample morphology. Using the gelatin embedding method allowed obtaining sections of imatinib-treated worms in high quality, enabling information-rich imaging analyses.

### Mass spectrometric profiling of *S. mansoni* for distinguishing worm sexes and for enabling imatinib detection

**Fig. 4:**
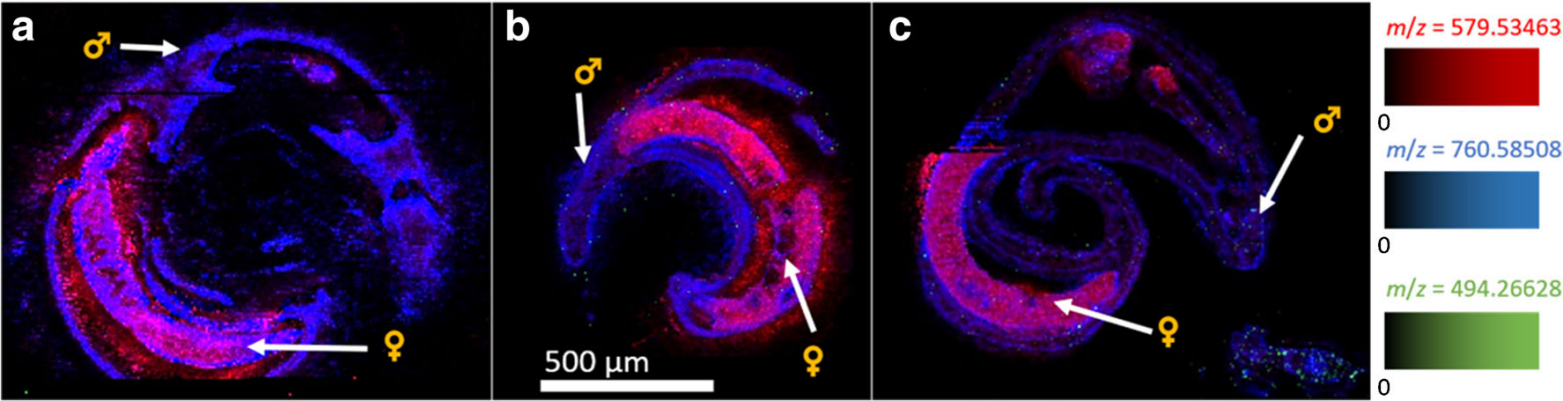
MSI images of *S. mansoni* couples of the control series. Depicted signals were *m*/*z* 760.585083 (blue, PC(34:1)), *m*/*z* 579.534686 (red, DG(34:0)), and *m*/*z* 494.266284 (green, imatinib). No imatinib or interfering signal at the same *m/z* value was detected in these untreated controls (i.e., no green pixels were obtained).Measured m/z values and errors: **a** 760.584974 (− 0.14 ppm); 579.534636 (− 0.09 ppm); 494.266445 (+ 0.32 ppm). **b** 760.584983 (− 0.13 ppm); 579.534620 (− 0.11 ppm); 494.265840 (− 0.9 ppm). **c** 760.584999 (− 0.11 ppm); 579.534594 (− 0.16 ppm); 494.266281 (− 0.01 ppm)

Cryosections of imatinib-treated *S. mansoni* were analyzed using high-resolution AP-SMALDI MSI. The mass spectra were obtained using pixel sizes between 5 and 9 μm. In Fig. 4, MSI images of three control measurements of worm couples without imatinib treatment are depicted. They were measured to assure that no interfering signal was detected at the *m*/*z* value of imatinib (no green pixels).

The displayed analytes represent two endogenous lipids in *S. mansoni*, the phosphatidylcholine (PC) PC(34:1) and the diacylglycerol (DG) DG(34:0), both assigned using the LIPID MAPS online database [39], in blue and red, respectively. No signal was detected at the *m/z* value of imatinib. The signal of PC(34:1) occurred abundantly in both animals with comparable intensities. DG(34:0) was mostly visible in the female, while the intensity in the male was low. For comparison with the optical images, please refer to the ESM (Fig. S2). The images of the individual color channels of the samples in Fig. 4 are depicted in the ESM (Fig. S3).

These first results exhibited signals representing lipid markers for *S. mansoni* females. The evaluation of signals specific for the female not only is of interest for basic research but also helps to facilitate male vs. female assignment in mass spectrometric images. One of these specific *m*/*z* signals was found at *m*/*z* 579.534639, which can be assigned to DG(34:0). The visible discrimination of male vs. female body parts became more complicated with rising imatinib concentration and incubation time, so the marker for the female worm was helpful to distinguish both worm sexes. Further marker signals were found for both male and female *S. mansoni* worms, as displayed in Table 1.

**Table 1:** Lipid markers for male and female *S. mansoni*, determined via assessment of MS images using AP-SMALDI MSI (PC = phosphatidylcholine; TG = triacylglycerol; DG = diacylglycerol; SM = sphingomyelin). Exemplary signal distribution for these ions can be seen in ESM Fig. S13

Sex of the adult worms	Measured *m*/*z*	Theoretical *m*/*z*	Error [ppm]	Annotated molecule	Sum formula of the ion
Female	768.587717	768.587763	− 0.06	PC(O-34:1)	[C_42_H_84_NO_7_P + Na]^+^
	746.605737	746.605818	− 0.11	PC(O-34:1)	[C_42_H_84_NO_7_P + H]^+^
	883.772498	883.772511	− 0.02	TG(52:1)	[C_55_H_104_O_6_ + Na]^+^
	579.534598	579.534686	− 0.15	DG(34:0)	[C_37_H_72_O_5_ + H – H_2_O]^+^
Male	785.652905	785.653102	− 0.25	SM(40:2)	[C_45_H_89_N_2_O_6_P + H]^+^
	783.637179	783.637452	− 0.35	SM(40:3)	[C_45_H_87_N_2_O_6_P + H]^+^
	703.574887	703.574852	0.05	SM(34:1)	[C_39_H_79_N_2_O_6_P + H]^+^
	731.606070	731.606200	− 0.11	SM(36:1)	[C_41_H_83_N_2_O_6_P + H]^+^
	807.634818	807.635047	− 0.28	SM(40:2)	[C_45_H_89_N_2_O_6_P + Na]^+^
	805.619087	805.619397	− 0.39	SM(40:3)	[C_45_H_87_N_2_O_6_P + Na]^+^

A previous study by Ferreira et al. [42] found markers for the male and female worms that differed from our study. This may be due to the fact that we investigated a Liberian strain of *S. mansoni*, while Ferreira et al. investigated two Brazilian strains. Also for the Brazilian strains, no identical sex markers were identified among the two strains, so the strains seemed to be rather different [42]. Furthermore, a 50-μm pixel size was used for image acquisition, while we used a smaller size of 5–9 μm, yielding a higher resolution and enabling discrimination of anatomical features, which might also contribute to the different outcome.

In the control samples, several endogenous lipid signals were found in the gelatin surrounding the animals. This may be due to the release of lipids from the worm to the outside of the animals in the culture medium. To prevent this leaking effect, the samples of the second measurement series were washed two times, once in plain culture medium before fixation in glutaraldehyde and once in water before the placement on the gelatin plateau. The additional washing steps after exposition to the drug and before the sectioning process prevented any contamination of the gelatin during sectioning. An example for this is given in Fig. 4. The first sample (a) was prepared using no washing steps, while the second sample (b) was prepared applying only the second washing step, resulting in no signal spreading. In the second measurement series with the lower concentration of imatinib, both washing steps were applied.

One general challenge is the analysis of non-flat samples using MSI at high lateral resolution, which can be overcome by using the pixelwise autofocusing function of the AP-SMALDI5 AF ion source [36, 37] to counteract height variations. Artifacts of this autofocusing operation may appear for highly reflective surfaces. One example of such artifacts is shown in Fig. 4c, where the red signal has a clear edge that was caused by problems with the autofocus. Such measurement artifacts were avoided using a higher amount of matrix solution, which was increased from 80 to 140 μL after the first eleven samples.

### AP-SMALDI MSI reveals imatinib distributions in different parasite tissues

**Fig. 5:**
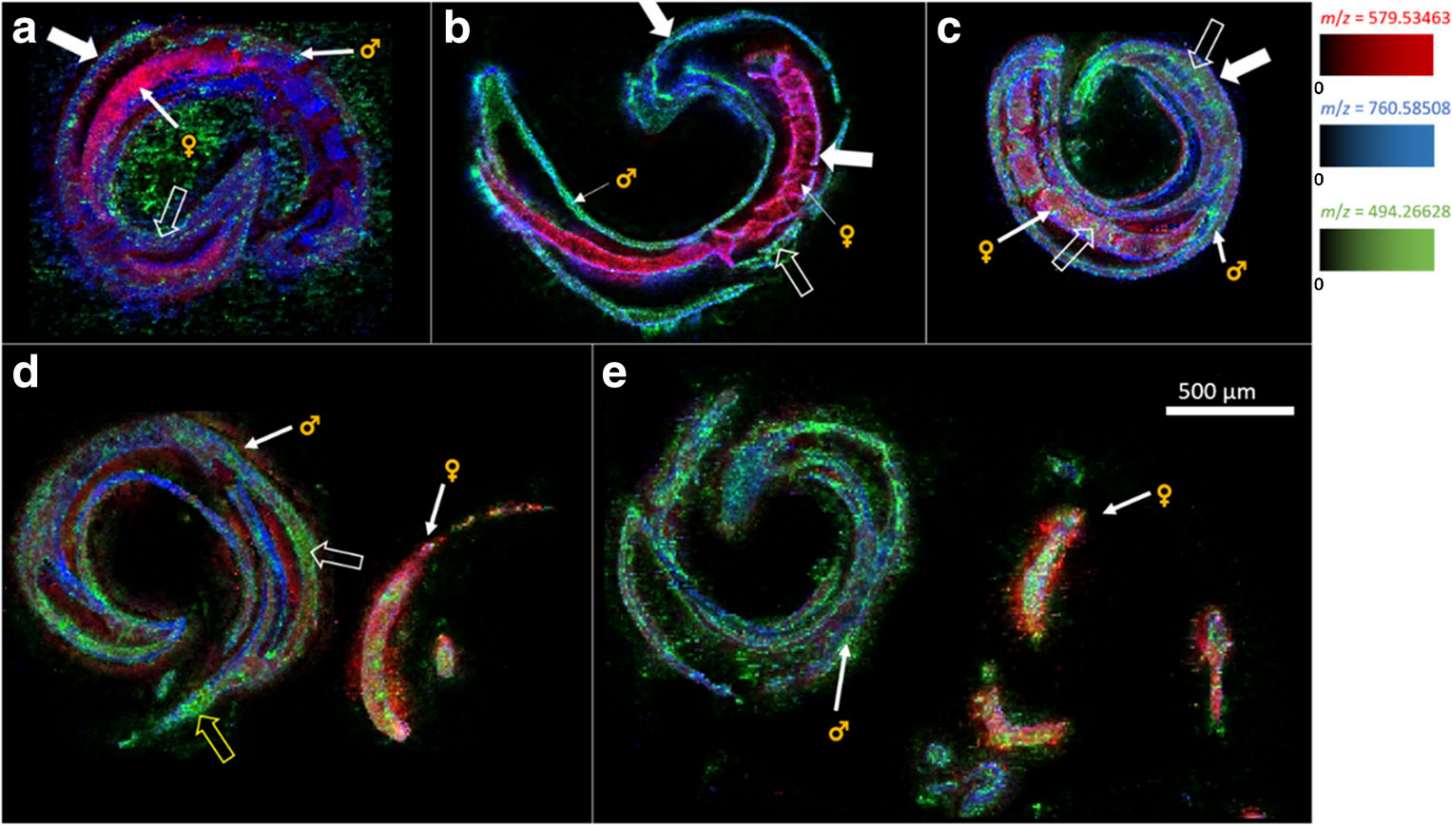
MSI images of *S. mansoni* couples using imatinib at a concentration of 100 μmol/L. Depicted analytes were *m*/*z* 494.266284 (green, imatinib), *m*/*z* 760.585083 (blue, PC(34:1)), and *m*/*z* 579.534686 (red, DG(34:0)); incubation times were 5 min (**a**), 20 min (**b**), 1 h (**c**), 4 h (**d**), and 12 h (**e**). For each time point, three worm couples were analyzed, of which one representative is shown here (filled arrows: tegument; unfilled arrows: intestine; unfilled, yellow arrow: esophagus). Measured m/z values and errors: **a** 494.266498 (+ 0.43 ppm), 760.584981 (− 0.13 ppm), 579.534576 (− 0.19 ppm); **b** 494.266309 (+ 0.05 ppm), 760.584937 (− 0.19 ppm), 579.534705 (+ 0.03 ppm); **c** 494.265444 (− 1.70 ppm), 760.584990 (− 0.12 ppm), 579.534598 (− 0.15 ppm); **d** 494.265425 (− 1.74 ppm), 760.584976 (− 0.14 ppm), 579.534588 (− 0.17 ppm); **e** 494.266480 (+ 0.40 ppm), 760.584997 (− 0.11 ppm), 579.534606 (− 0.14 ppm)

The first series of MSI measurements was performed with couples treated with imatinib at a concentration of 100 μmol/L (Fig. 5). The same *m*/*z* values as in the control samples (Fig. 4) were selected for the images.

The lipid signals showed the same distributions as in the control samples. Males and females were easily differentiated using the female-indicative red signal of DG(34:0). The blue signal of PC(34:1) was detectable in both animals with a similar distribution characteristics. As early as 5 min after exposure to imatinib (Fig. 5a), the drug was detected at the tegumental surface but also as faint signals within the male, while there was no signal inside the female at this time point. Imatinib signals were also detected from outside the couple, which may have resulted from a smearing effect of the sample during sectioning, as it has occasionally been observed for lipids. Imatinib was mainly detected in the intestine and tegument of the male. A similar distribution was found after 20 min of treatment but with a higher signal intensity within the male. In addition, imatinib appeared at the surface of the female (Fig. 5b). This coincided with the presence of imatinib at the ventral surface of the male which faces the female body. After 1 h (Fig. 5c), imatinib was detected in the female’s intestine (Fig. 5d). At this time point, the male sections showed signals inside the head area, which is indicative for the esophagus, suggesting an oral uptake route of imatinib into the digestive tract. After longer exposure times of 4 h and 12 h, imatinib was found to be distributed throughout the worms; however, the couples separated as a consequence of drug activity (Fig. 5d, e). After 12 h (Fig. 5e), the worms were very fragile, and the sections showed several cracks due to drug effects. Please refer to the ESM (Figs. S4, S8, and S9) for comparison with optical images, for individual color channel images of Fig. 5, and for complete MSI results.

AP-SMALDI MSI allowed the detection of imatinib in various parasite tissues and revealed distinct uptake kinetics for male and female worms.

### Imaging *S. mansoni* couples exposed to a lower concentration of imatinib confirmed the uptake route while stabilizing the pairing status

**Fig. 6:**
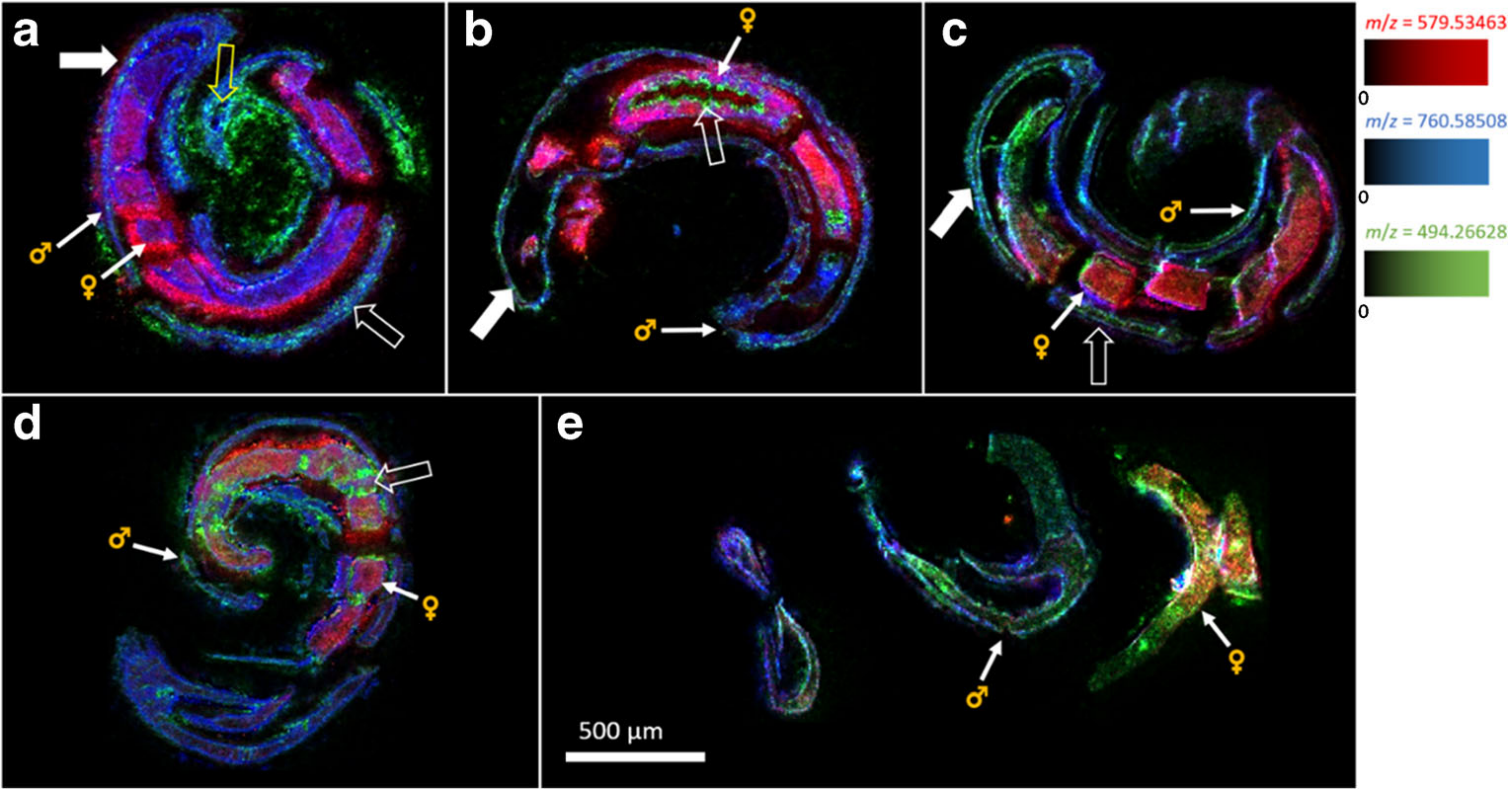
MSI images of *S. mansoni* couples at an imatinib concentration of 20 μmol/L. Depicted analytes were *m*/*z* 494.266284 (green, imatinib), *m*/*z* 760.585083 (blue, PC(34:1)), and *m*/*z* 579.534686 (red, DG(34:0)); incubation times were 20 min (**a**), 1 h (**b**), 4 h (**c**), 12 h (**d**), and 24 h (**e**). For each time point, two worm couples were analyzed, of which one representative for each time point is shown here (filled arrows: tegument; unfilled arrows: intestine; unfilled, yellow arrow: esophagus). Measured m/z values and errors: **a** 494.266554 (+ 0.55 ppm), 760.584979 (− 0.14 ppm), 579.534635 (− 0.09 ppm); **b** 494.266600 (+ 0.64 ppm), 760.584924 (− 0.02 ppm), 579.534677 (− 0.21 ppm); **c** 494.266562 (+ 0.56 ppm), 760.584908 (− 0.23 ppm), 579.534785 (+ 0.17 ppm); **d** 494.266546 (+ 0.53 ppm), 760.584994 (− 0.12 ppm), 579.534639 (− 0.08 ppm); **e** 494.266481 (+ 0.40 ppm), 760.584981 (− 0.13 ppm), 579.534695 (+ 0.01 ppm)

To overcome the imatinib-induced separation of couples while imaging the uptake route of imatinib, we conducted a second measurement series for paired worms treated with a lower imatinib concentration of 20 μmol/L (Fig. 6). This was the highest possible concentration that did not affect pairing stability within 12 h, but which already induced clear effects on tissue morphology. The latter included gut dilatations which were detected by bright-field microscopy (ESM Fig. S7 d), indicating the uptake and presence of imatinib. The *m*/*z* channels depicted in Fig. 6 are the same as in the control samples and the samples of the high-concentration treatment group (Figs. 4 and 5).

Imatinib, applied with 20 μmol/L concentration, was detectable almost as well as at a concentration of 100 μmol/L and showed similar kinetics. After an incubation time of 20 min (Fig. 6a), imatinib signals occurred in the esophagus, intestine, and tegument of the male, while detectable imatinib signals were absent in the female. From 1 h and onwards (Fig. 6b–e), imatinib was detected at the surface and within the internal structures of both sexes. Higher signal intensities were detected in the intestines. The example shown in Fig. 6b exhibits a strong imatinib signal covering the inner surface of the intestine, i.e., the gastrodermis, while no imatinib signal was observed in the gut lumen. The worms separated after an incubation time of 24 h (**e**), the tissue became brittle and caused more sectioning artifacts. Imatinib was evenly distributed in both animals at this time point. Please refer to the ESM (Figs. S5, S10, and S11) for comparison with optical images, for individual color channel images of Fig. 6, and for complete MSI results.

Overall, the results of this measurement series with 20 μmol/L imatinib concentration were similar to the results of the high-concentration treatment group using 100 μmol/L imatinib, with the advantage that pairing stability was maintained for 12 h despite imatinib treatment. The presence of imatinib in the female intestine found from 1 h onwards correlated with the damage of the gastrodermis, observed by bright-field microscopy.

### AP-SMALDI MSI revealed possible routes of drug uptake

The various imaging series suggested that *S. mansoni* worms incorporate imatinib via an oral route. The drug was found at the oral sucker, at the esophagus, and further down in the intestine (e.g., Fig. 5d). From there, imatinib is presumably resorbed by the gastrodermis, the inner layer of the intestine, which showed high intensities of the imatinib signal (e.g., Fig. 6b). A second, additional route of uptake may occur via the tegument, the outer, physiologically active surface layer of schistosomes. With respect to the two sexes, we observed an interesting difference in the kinetics of the imatinib distribution in the tissue. For male worms, the uptake started right after imatinib exposition, within 5 min of incubation time. Imatinib signal intensities were low after 5 min (average intensity NL = 7.18 ∙ 10^2^) and two times as high after 20 min (average intensity NL = 1.64 ∙ 10^3^) at a concentration of 100 μmol/L. In contrast, imatinib signals started to occur in the tegument of females not before 20 min of incubation, and it took 60 min until imatinib was detected in inner tissues (Figs. 5c and 6b). This suggests a delay of imatinib uptake in the female compared to the male, which could be explained by the fact that the female lives in the gynaecophoric canal of the male, where it is partly protected by the male’s body towards the environment.

In conclusion, AP-SMALDI MSI provided the first evidence for uptake routes of imatinib into *S. mansoni* couples.

### Identification of an ovary-related lipid marker to study drug distribution

**Fig. 7:**
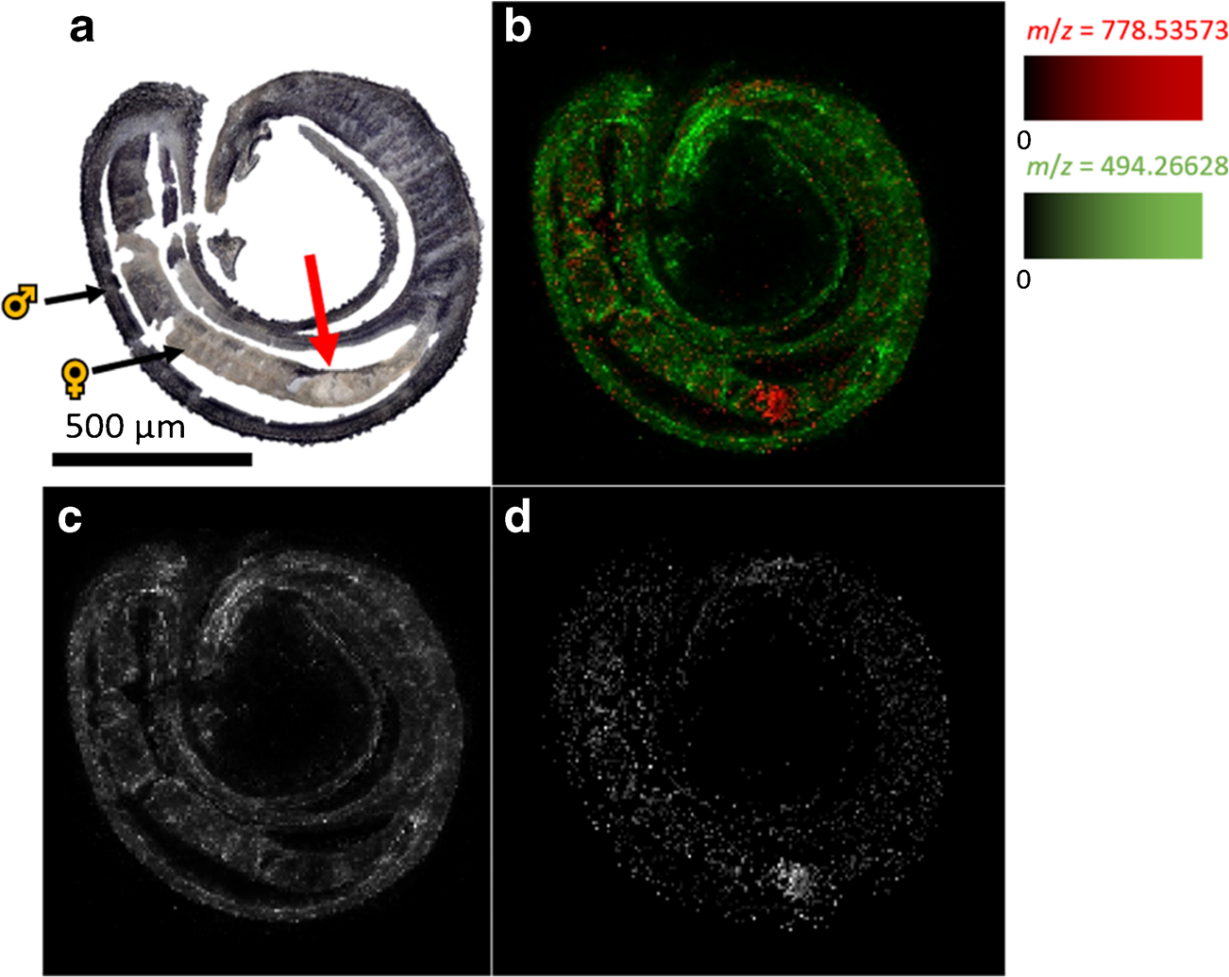
Mapping of imatinib and an ovary-related lipid marker. **a** Optical image of a tissue section of an *S. mansoni* couple, 100 μmol/L imatinib, 1 h incubation time, arrow marks the ovary; **b** MSI image of the *S. mansoni* couple tissue section, *m*/*z* 494.266284 (green, imatinib), *m*/*z* 778.535728 (red, PC(34:3)); **c** MSI image of the *S. mansoni* couple, *m*/*z* 494.266284 (imatinib), corresponding to the green channel in **b**; **d** MSI image of the *S. mansoni* couple, *m*/*z* 778.535728 (PC(34:3)), corresponding to the red channel in **b**. Measured m/z values and errors: 494.265444 (− 1.70 ppm), 778.535266 (− 0.59 ppm), 579.534635 (− 0.09 ppm)

Discriminating the two sexes in a worm couple can be challenging when interpreting MSI images. This was solved by the identification of an analyte signal which occurred predominantly in paired females (Fig. 4). The discrimination of specific organs within each worm can be another challenging task. This, however, may become of interest when correlations to drug-induced tissue damages are examined. The lipid at *m*/*z* 778.538133, depicted as the red color channel in Fig. 7, showed highest intensities in the ovary. This lipid was identified as PC(34:3) according to the lipid maps online database [39]. The intensity of imatinib was considerably lower at this location. Thus, the uptake of imatinib into the ovary was less efficient than into the rest of the female worm.

Comparing the results from CLSM and AP-SMALDI MSI showed that the observed morphological changes correlate well with the presence of imatinib. Imatinib was found in the intestine of male and female worms after 5 min and 1 h, respectively, which caused detectable disintegration of gastrodermal tissue after 4 h. For female ovaries, however, no considerable amounts of imatinib were detectable even after 12 h, despite clear ovarian tissue destruction after an incubation time of 24 h at the same imatinib concentration [15]. Besides possible differences in tissue-specific limits of detection of ovaries and other worm organs, this might be explained by a high sensitivity of the ovaries even to low, hardly detectable concentrations of imatinib. About 30% of the schistosomal ovary is made up of proliferative stem cells [43], and stem cells are known to be particularly sensitive to certain compounds [44]. Furthermore, the presumed targets of imatinib in *S. mansoni*, the Abl kinases 1 and 2, were found to be expressed in considerably higher levels in the ovary compared to the gastrodermis [15], which might account for a higher ovarian drug sensitivity. In addition or alternatively to these direct effects, the ovary might also be affected indirectly, for instance by action of imatinib on surrounding tissues with downstream effects on the ovary.

### Identification of drug metabolites within the parasite

**Fig. 8:**
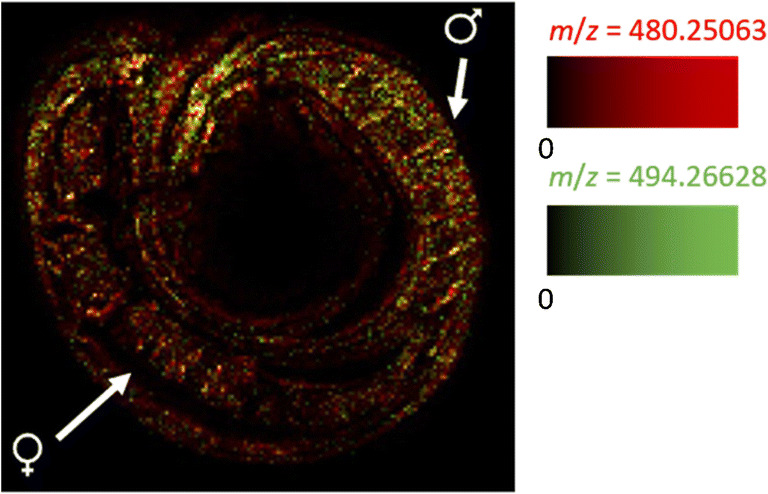
MSI image of an *S. mansoni* couple tissue section, 100 μmol/L imatinib, 1 h incubation time, *m*/*z* 494.266284 (red, imatinib), and *m*/*z* 480.250634 (green, N-desmethyl imatinib). One representative of the 26 measured worm samples is shown. Measured m/z values and errors: 494.265444 (− 1.70 ppm), 480.249815 (− 1.71 ppm)

N-Desmethyl-imatinib is the main bioactive metabolite of imatinib found in human patients. Although it is less active than imatinib, it might still contribute to drug activity [45]. We therefore investigated whether schistosomes are capable of metabolizing imatinib, and if yes, after which time the metabolite is detectable. In Fig. 8, the MS image of a *S. mansoni* couple, treated for 1 h with an imatinib concentration of 100 μmol/L, is shown. The displayed signals are imatinib (*m*/*z* 494.266284) in red and N-desmethyl imatinib (*m*/*z* 480.250634) in green. Both signals showed a similar distribution throughout the bodies of male and female worms. In the measurement series of the lower-concentration treatment group (20 μmol/L), the metabolite could not be detected in all samples (ESM Fig. S12).

This result strongly suggests that imatinib is metabolized by *S. mansoni* to the same metabolite as found in humans. The signal intensities of the metabolite were much lower than of imatinib at all time points. Furthermore, the intensity ratios of N-desmethyl imatinib and imatinib increased from 1.8 ∙ 10^−3^ after 20 min to 3.29 ∙ 10^−3^ at 1 h and 3.97 ∙ 10^−3^ at 4 h (ESM Fig. S12). These findings suggest a low rate of metabolization and an accumulation of the metabolite over time. As the metabolite is capable of inhibiting cell proliferation and inducing apoptosis in human cells [45, 46], it might very well contribute to the tissue destruction found in imatinib-treated *S. mansoni*.

## Conclusion

The distribution of imatinib in sections of *S. mansoni* can be well assessed by AP-SMALDI MSI. Two experimental series with different imatinib concentrations (100 μmol/L and 20 μmol/L) were performed using incubation times ranging from 5 min to 24 h. The data give indications that imatinib is taken up via the oral route and perhaps also via the tegumental surface of the worms. The male shields the female in the gynaecophoric canal, which might explain why imatinib was not detectable in the female after short incubation periods. Signal intensities of imatinib in the ovary of the female were lower compared to the rest of the worm. This may be due to lower uptake of substances into the ovary than into other organs. At the level of differentially occurring lipid classes, distinctions between male and female *S. mansoni* as well as the allocation of organs can be achieved.

This is the first time that the distribution of a drug was directly investigated in a parasite, the human pathogen *S. mansoni*, using AP-SMALDI MSI. Comparison to previous studies of the imatinib effects on the morphology of adult *S. mansoni* showed high congruence. Furthermore, as an untargeted approach, the applied technique opened the possibility of determining metabolites, such as N-desmethyl-imatinib.

AP-SMALDI MSI was established as a technique for studying kinetics and drug metabolism in a multicellular parasite. With respect to the urgent need of finding alternative treatments against pathogens threatening not only human but also animal health in a one-health context [47, 48], methods such as AP-SMALDI MSI can be considered as helpful tools. With this technique, it is possible to study not only the effects of repurposed drugs but also novel compounds in schistosomes as well as other pathogens of the NTD spectrum and beyond, if in vitro culture systems are available.

## Supplementary information

ESM 1 (PDF 2296 kb)

## Data Availability

MS imaging data is available through the Metaspace online data repository (https://metaspace2020.eu).
