# Supplementary material for: High-resolution AP-SMALDI MSI as a tool for drug imaging in Schistosoma mansoni
Source: Anal Bioanal Chem. 2021 Mar 15;413(10):2755–66. doi: 10.1007/s00216-021-03230-w (PMC8007518; doi:10.1007/s00216-021-03230-w)
Supplement: Supplementary file 1 — (PDF 2296 kb) [file 216_2021_3230_MOESM1_ESM.pdf]

High-resolution AP-SMALDI MSI as a tool for drug imaging in *Schistosoma mansoni*

A. Mokosch[1], S. Gerbig[1], C. G. Grevelding[2], S. Haeberlein[2*], B. Spengler[1*]

[1]Institute of Inorganic and Analytical Chemistry, Justus Liebig University Giessen, Giessen, Germany

[2]Institute of Parasitology, BFS, Justus Liebig University Giessen, Giessen, Germany

Corresponding author:
Bernhard.Spengler@anorg.Chemie.uni-giessen.de

* joint senior authorship

SUPPLEMENTARY INFORMATION

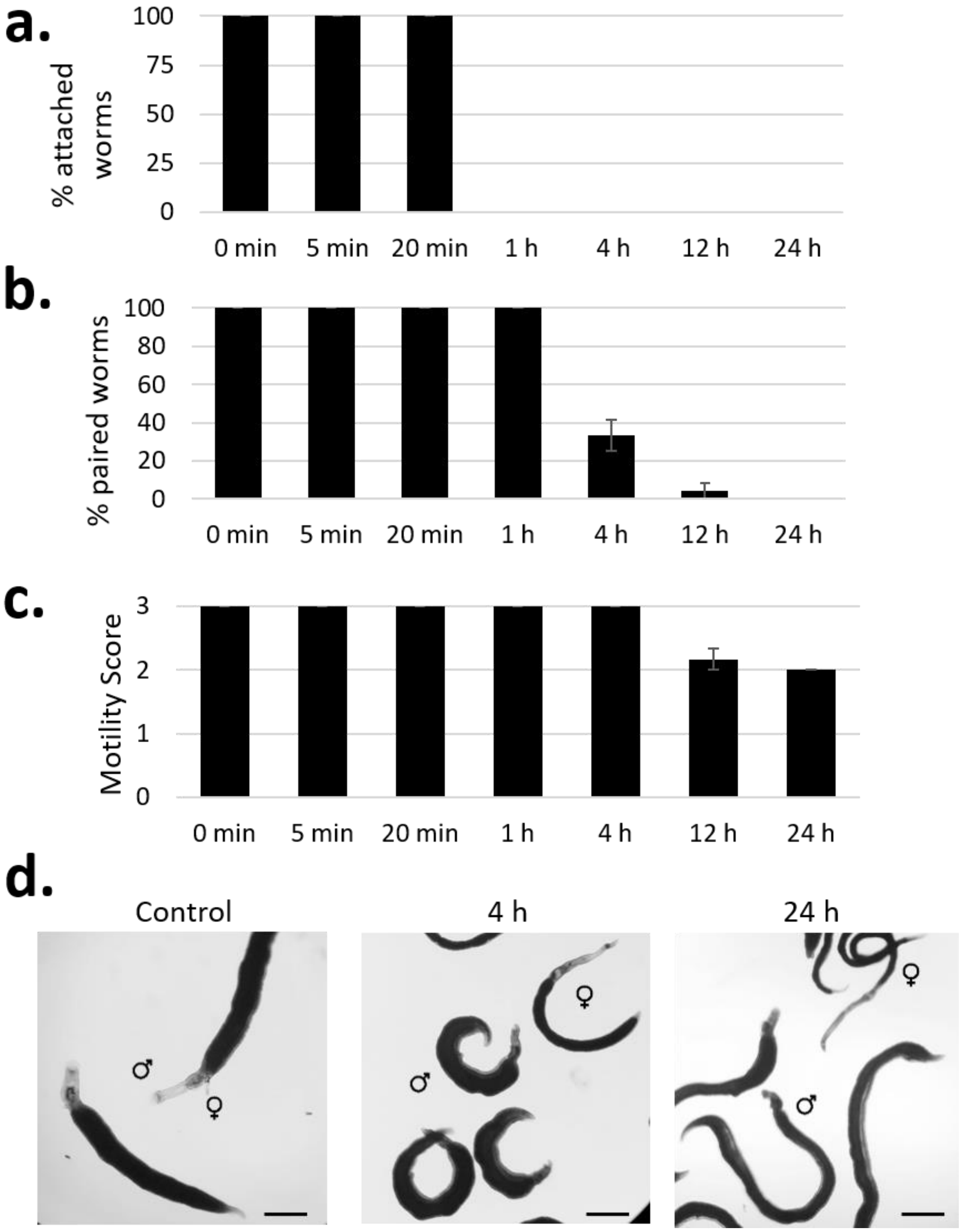

Fig. S1: Phenotypes of worm couples after various exposure times to 100 µmol/L imatinib, used for AP-SMALDI MSI. (a.) Fitness of worms was determined by the percentage of vital worms attaching to the culture well. After 1 h of drug treatment, all worms were detached. (b.) Percentage of paired worms. After 4 h, male and female worms started to separate. (c.) Motility score. A first reduction of worm motility was noticed after 12 h exposure. The mean of two independent experiments with ten worm couples per experiment is shown. (d.) Representative images from bright-field microscopy of an untreated (control) worm couple and worm couples treated for 4 h and 24 h with 100 µmol/L imatinib. Scale bars: 0.5 mm.

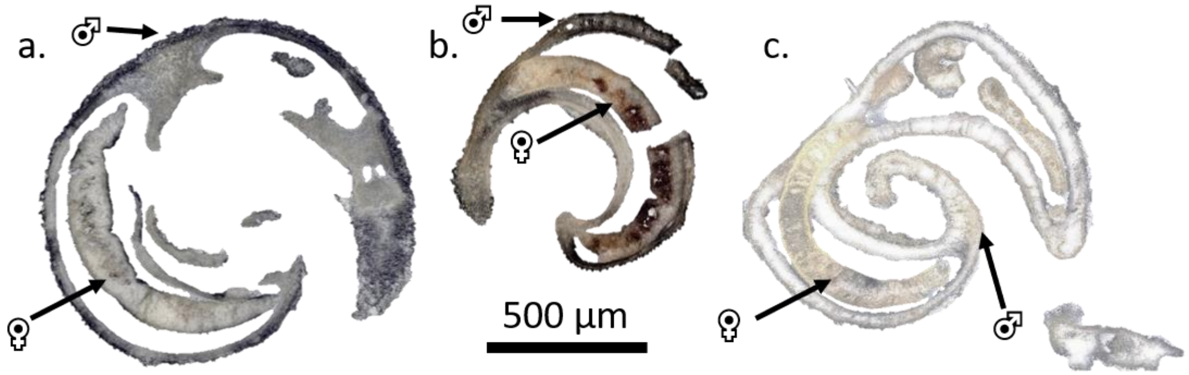

Fig. S2: Optical images of sections of *S. mansoni* couples without imatinib treatment, a, b and c correspond to Fig. 4. Male and female worms were visible in all samples.

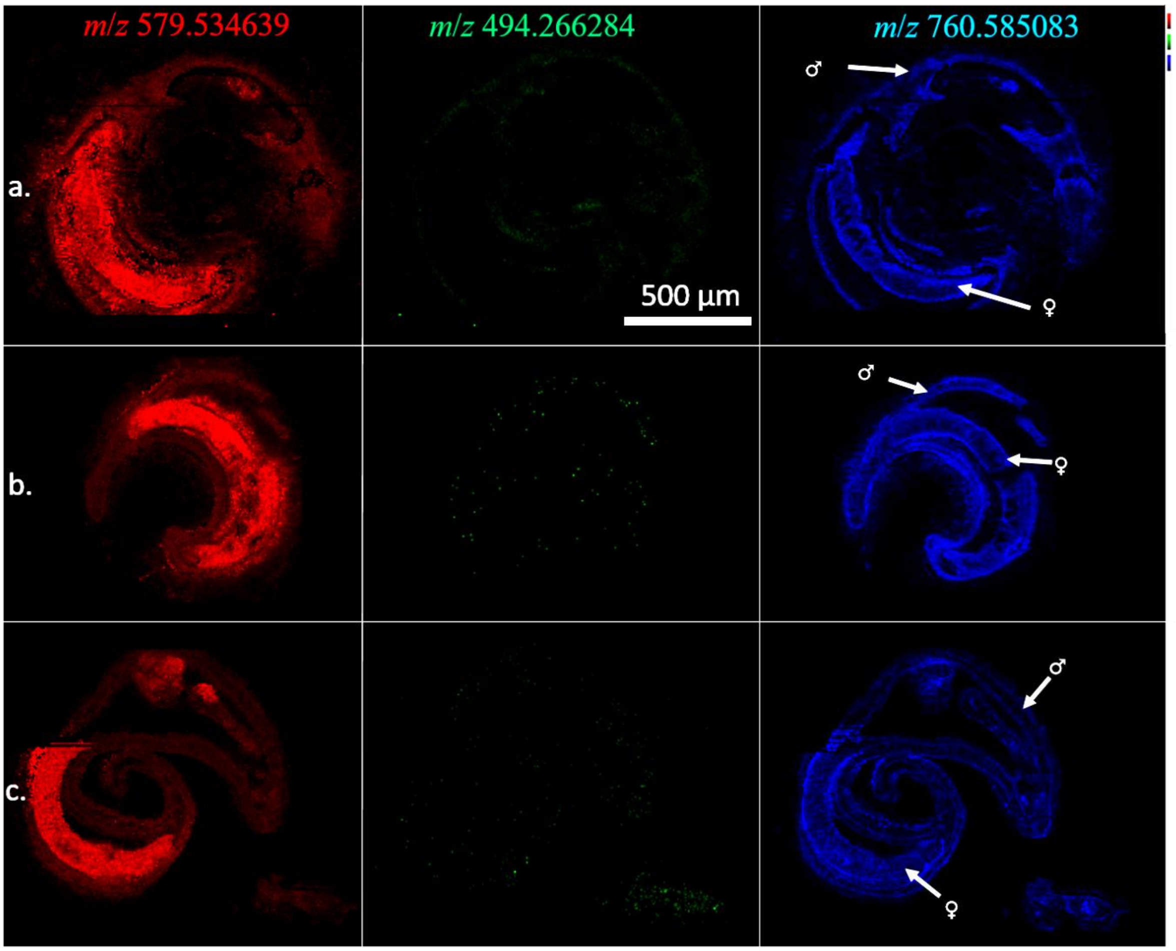

Fig. S3: Individual color channels of AP-SMALDI MS images: sections of the control samples a, b, and c (according to Fig. 4); *m/z* 579.534636 ((a.), 579.534620 (b.), 579.534594 (c.); red, DG(34:0); theoretical *m/z*: 579.534686; error: -0.09 ppm (a.), -0.11 ppm (b.), -0.16 ppm (c.)), *m/z* 494.266445 ((a.), 494.265840 (b.), 494.266281 (c.); green, imatinib; theoretical *m/z*: 494.266284; error: 0.32 ppm (a.), -0.90 ppm (b.), -0.01 ppm (c.)), *m/z* 760.584974 ((a.), 760.584983 (b.), 760.584999 (c.); blue, PC(34:1); theoretical *m/z*: 760.585083; error: -0.14 ppm (a.), -0.13 ppm (b.), -0.11 ppm (c.)).

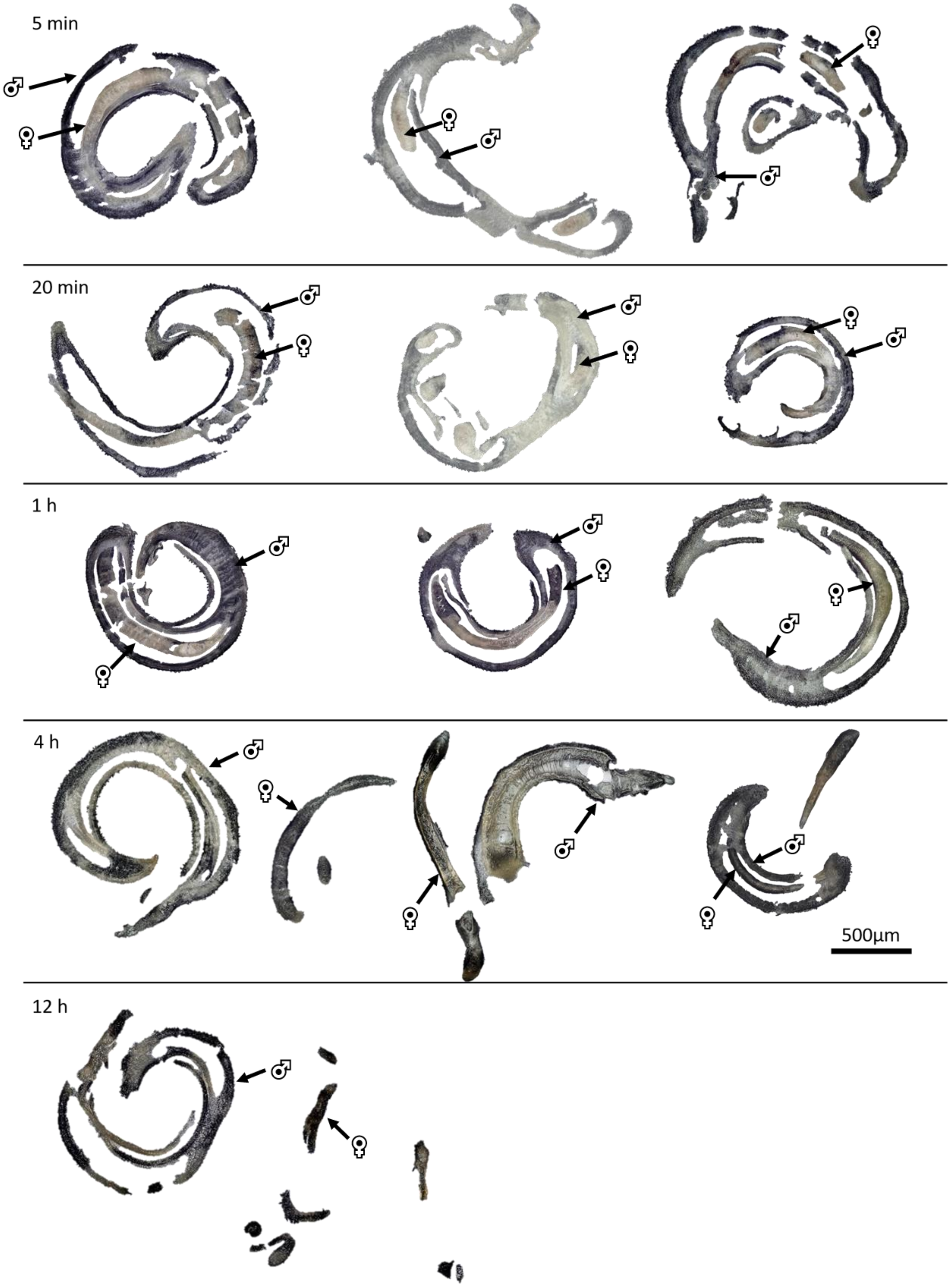

Fig. S4: Optical images of sections of *S. mansoni* couples, treated with imatinib (100 µmol/L). The first picture of each row matches with the MS image for the corresponding incubation time in Fig. 5. Male and female worms were visible in all samples. After 4 h, the couples separated.

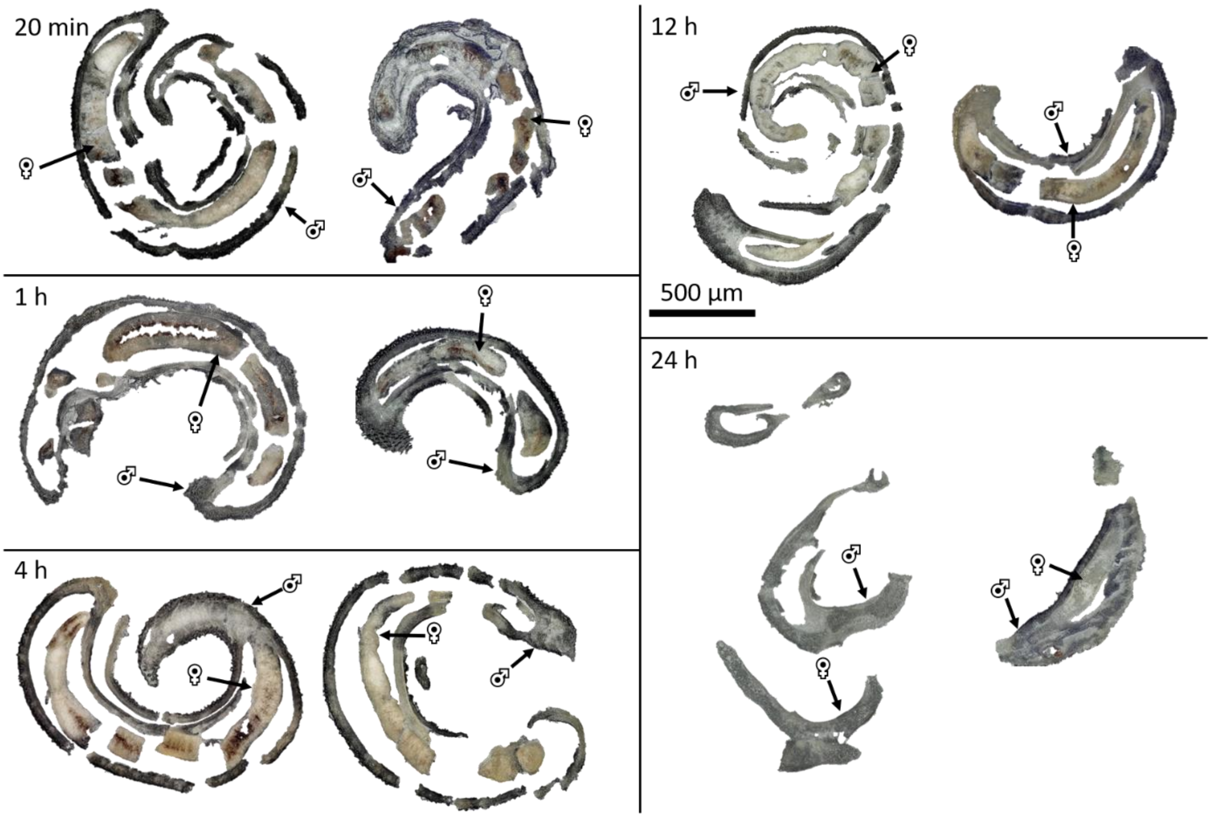

Fig. S5: Optical images of sections of *S. mansoni* couples, treated with a lower concentration of imatinib (20 µmol/L). The first picture of each time point matches with the MS image for the corresponding incubation time in Fig. 6. Male and female worm were visible in all samples. After 24 h, the couples separated.

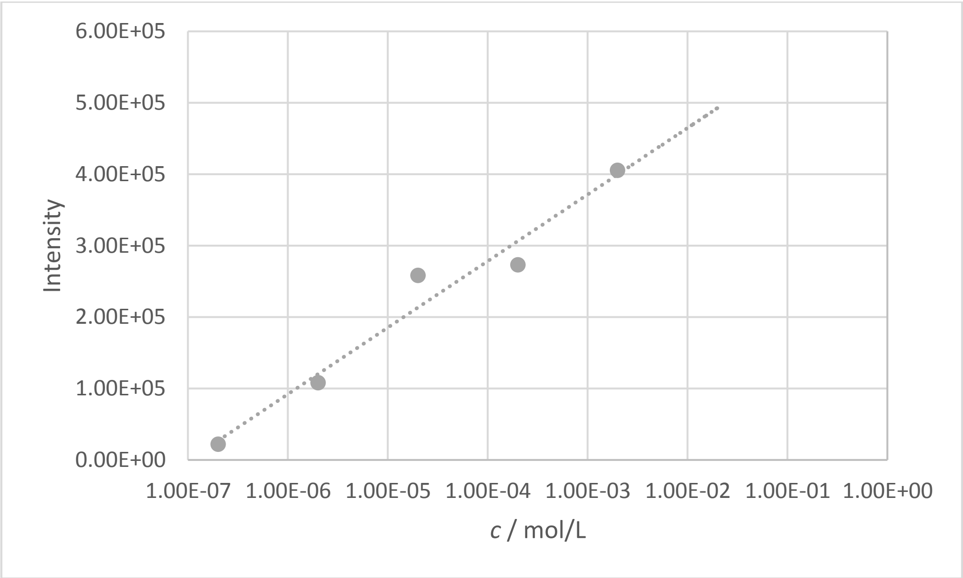

Fig. S6: Measurement series of the imatinib standard. The solutions of the dilution series were applied to a slide and measured using MALDI-MS. The intensity of the imatinib signal was plotted against the concentrations used.

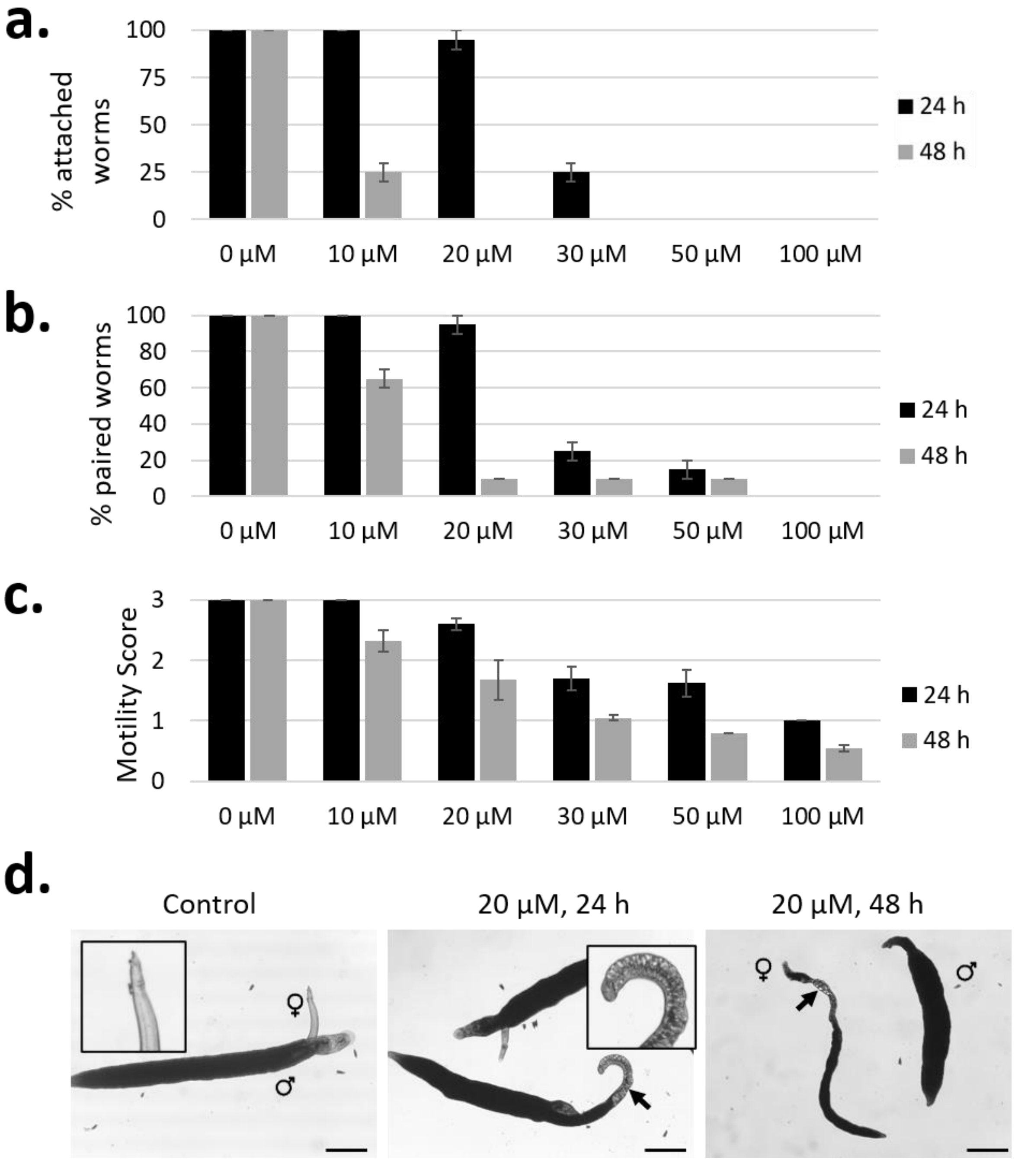

Fig. S7: Titration of imatinib to determine the optimal concentration for studying paired worms. (a.) Fitness of worms was determined by the percentage of vital worms attaching to the culture well. After 24 h and 20 µmol/L imatinib, almost all worms were still attached; higher concentrations caused detachment. (b.) Percentage of paired worms. After 24 h, almost all worms were still paired when incubated in 20 µmol/L imatinib; higher concentrations caused the separation of worm couples. (c.) Motility score. A first reduction of worm motility was noticed after 24 h exposure to 20 µmol/L imatinib. Mean values of two independent experiments with ten worm couples per experiment are shown. (d.) Representative images from bright-field microscopy of an untreated (control) worm couple and imatinib-treated worm couples after 24 h and 48 h. Arrows indicate gut dilatations in the female worms. Scale bar: 0.5 mm.

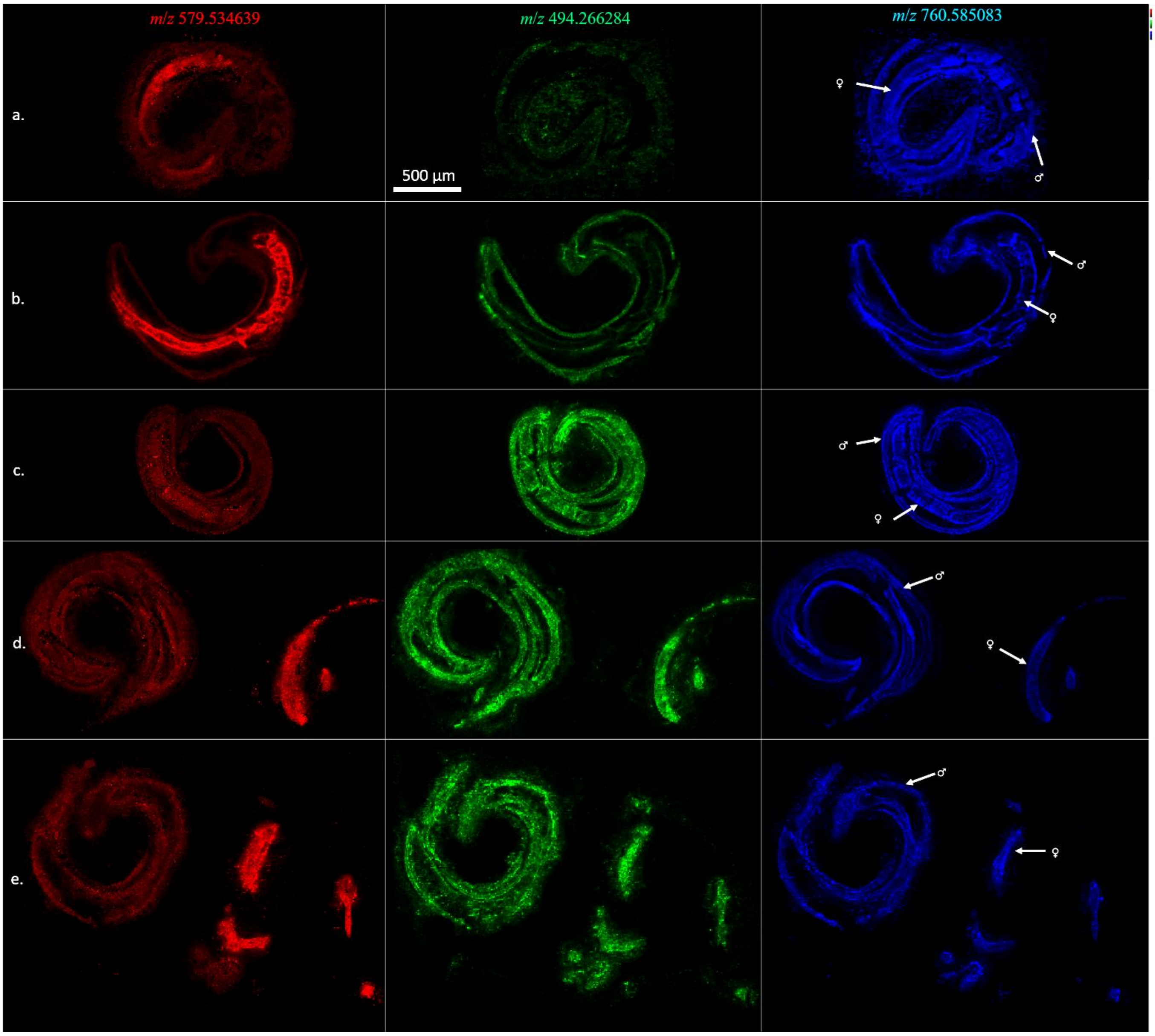

Fig. S8: Individual color channels of AP-SMALDI MS images: sections of couples a to e, following imatinib treatment (100 µmol/L) (according to Fig. 5); *m*/*z* 579.534576 ((a.), 579.534705 (b.), 579.534598 (c.), 579.534588 (d.), 579.534606 (e.); red, DG(34:0); theoretical *m*/*z*: 579.534686; error: -0.19 ppm (a.), 0.03 ppm (b.), -0.15 ppm (c.), -0.17 ppm (d.), -0.14 ppm(e.)), *m*/*z* 494.266498 ((a.), 494.266309 (b.), 494.265444 (c.), 494.265425 (d.), 494.266480 (e.); green, imatinib; theoretical *m*/*z*: 494.266284; error: 0.43 ppm (a.), 0.05 ppm (b.), -1.70 ppm (c.), -1.74 ppm (d.), 0.40 ppm (e.)), *m*/*z* 760.584981 ((a.), 760.584937 (b.), 760.584990 (c.), 760.584976 (d.), 760.584997 (e.); blue, PC(34:1); theoretical *m*/*z*: 760.585083; error: -0.13 ppm (a.), -0.19 ppm (b.), -0.12 ppm (c.), -0.14 ppm (d.), -0.11 ppm (e.)). Imatinib treatment was applied for 5 min (a), 20 min (b), 1 h (c), 4 h (d) and 12 h (e). Images of the imatinib signal (green) show the development of imatinib uptake with treatment time: very low after 5 minutes (a), only in tegument of the male after 20 minutes (b), in male and female after 1 hour (c) and strong in decoupled male and female after 4 and 12 hours (d and e).

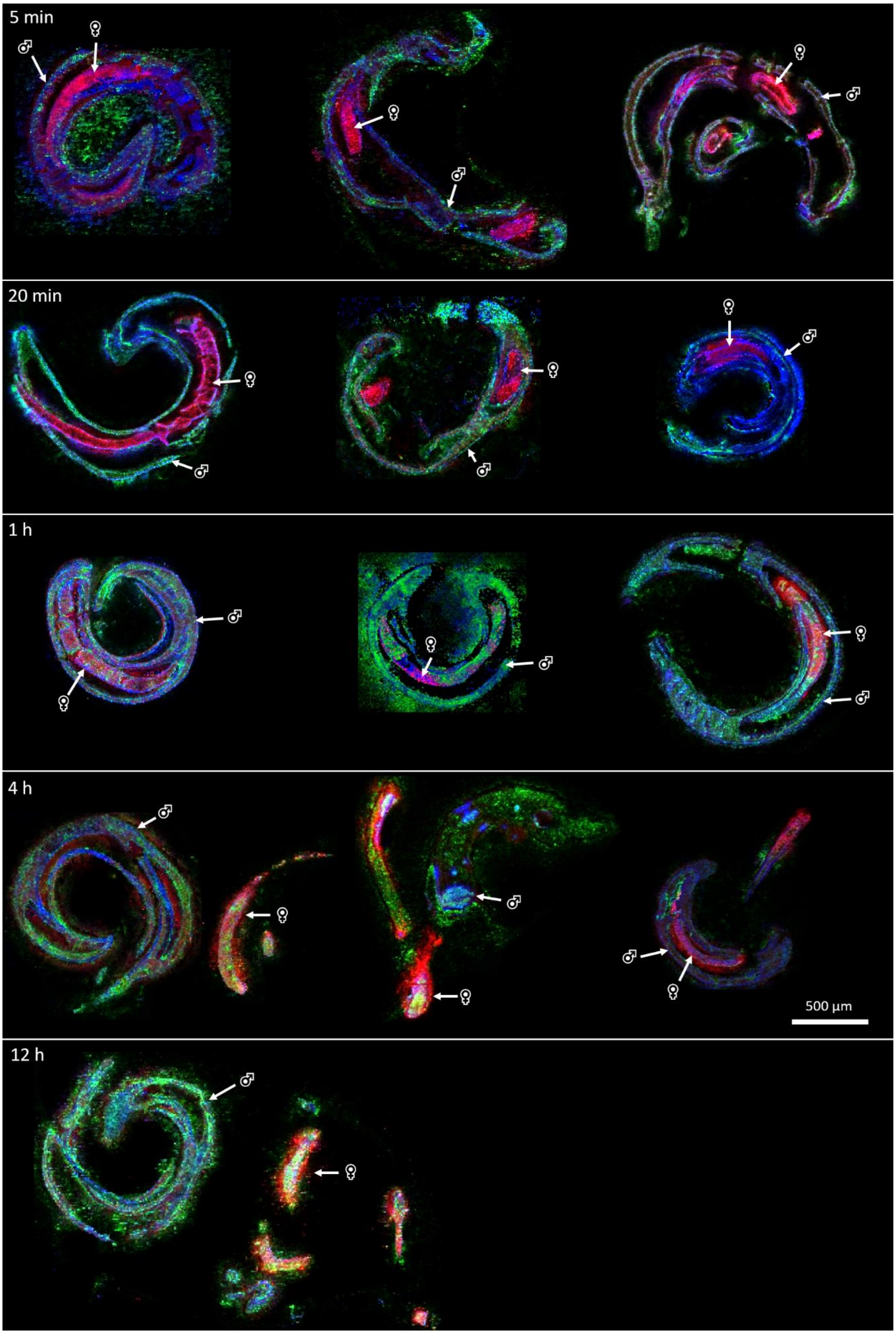

Fig. S9: AP-SMALDI MS images of sections of *S. mansoni* couples after imatinib treatment (100 µmol/l). MS images match with the optical images for the corresponding incubation time in supplementary Fig. S4. Visualized are *m/z* 494.266456 ((5 min), 494.266175 (20 min), 494.265994 (1 h), 494.266132 (4 h), 494.266480 (12 h); green, imatinib; theoretical *m/z*:

494.266284; error: 0.35 ppm (5 min), -0.22 ppm (20 min), -0.59 ppm (1 h), -0.31 ppm (4 h), 0.40 ppm (12 h)), *m*/*z* 760.584980 ((5 min), 760.584951 (20 min), 760.585003 (1 h), 760.584991 (4 h), 760.584997 (12 h); blue, PC(34:1); theoretical *m*/*z*: 760.585083; error: -0.14 ppm (5 min), -0.17 ppm (20 min), -0.11 ppm (1 h), -0.12 ppm (4 h), -0.11 ppm (12 h)), and *m*/*z* 579.534624 ((5 min), 579.534793 (20 min), 579.534765 (1 h), 579.534604 (4 h), 579.534606 (12 h); red, DG(34:0); theoretical *m*/*z*: 579.534686; error: -0.11 ppm (5 min), 0.18 ppm (20 min), 0.14 ppm (1 h), -0.14 ppm (4 h), -0.14 ppm(12 h)). The experimental values are the average of the samples shown for the respective incubation times.

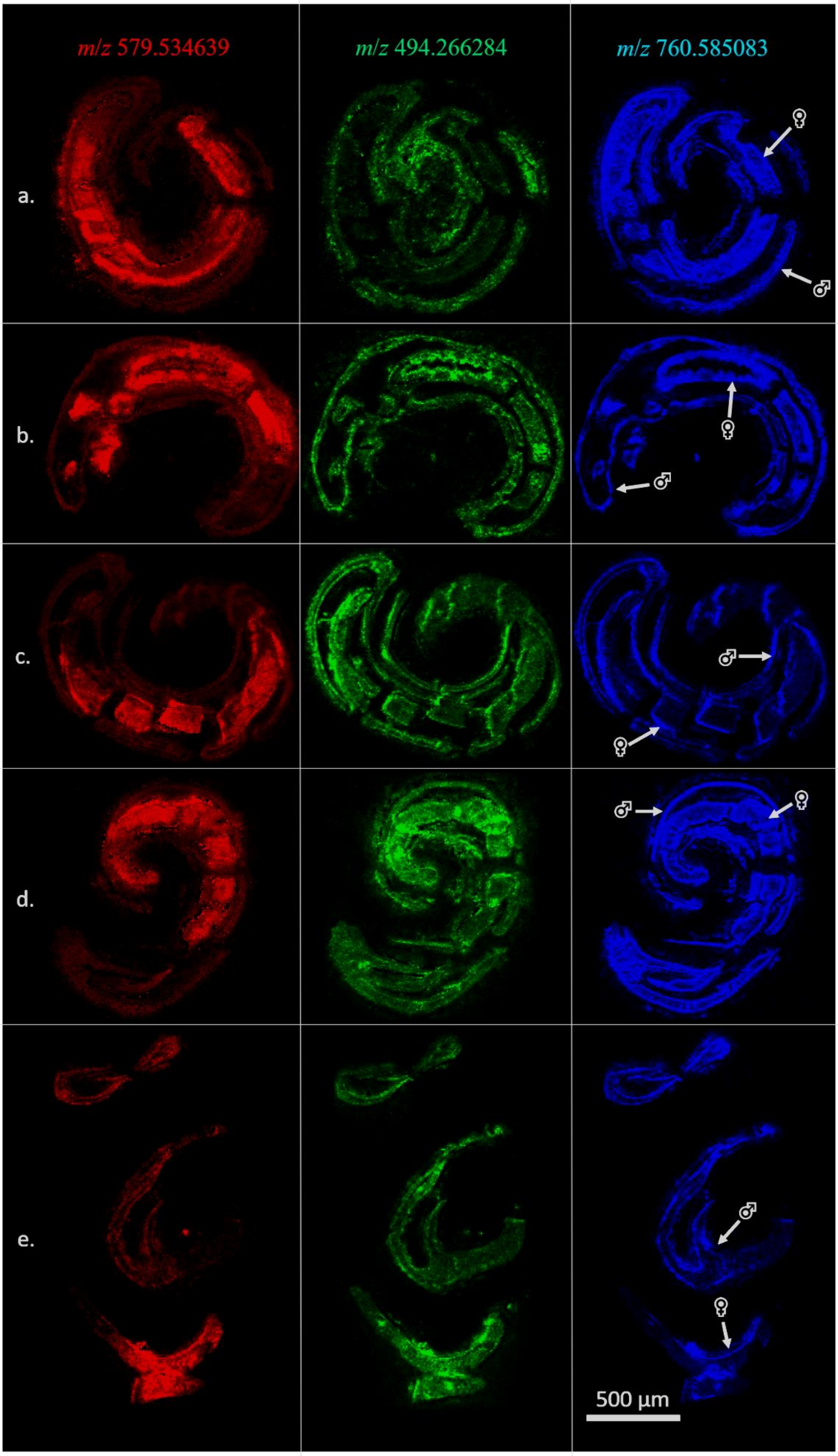

Fig. S10: Individual color channels of AP-SMALDI MS images: sections of *S. mansoni* couples a to e, treated with 20 µmol/L imatinib (according to Fig. 6); *m/z* 579.534635 ((a.), 579.534677 (b.), 579.534785 (c.), 579.534639 (d.), 579.534695 (e.); red, DG(34:0); theoretical *m/z*: 579.534686; error: -0.09 ppm(a.), -0.21 ppm (b.), 0.17 ppm (c.), -0.08 ppm (d.), 0.01 ppm (e.)),

m/z 494.266554 ((a.), 494.266600 (b.), 494.266562 (c.), 494.266546 (d.), 494.266481 (e.); green, imatinib; theoretical *m/z*: 494.266284; error: 0.55 ppm (a.), 0.64 ppm (b.), 0.56 ppm (c.), 0.53 ppm (d.), 0.40 ppm (e.)), and *m/z* 760.584979 ((a.), 760.584924 (b.), 760.584908 (c.), 760.584994 (d.), 760.584981 (e.); blue, PC(34:1); theoretical *m/z*: 760.585083; error: -0.14 ppm (a.), -0.02 ppm (b.), -0.23 ppm (c.), -0.12 ppm (d.), -0.13 ppm (e.)). Imatinib treatment was applied for 20 min (a), 1 h (b), 4 h (c), 12 h (d), and 24 h (e). Images of the imatinib signal (green) show the development of imatinib uptake with treatment time: only in tegument of the male after 20 minutes (a), in male and female after 1 hour (b) with increasing intensities after 4 hours (c) and strong and unspecific in degraded and decoupled male and female after 12 and 24 hours (d and e).

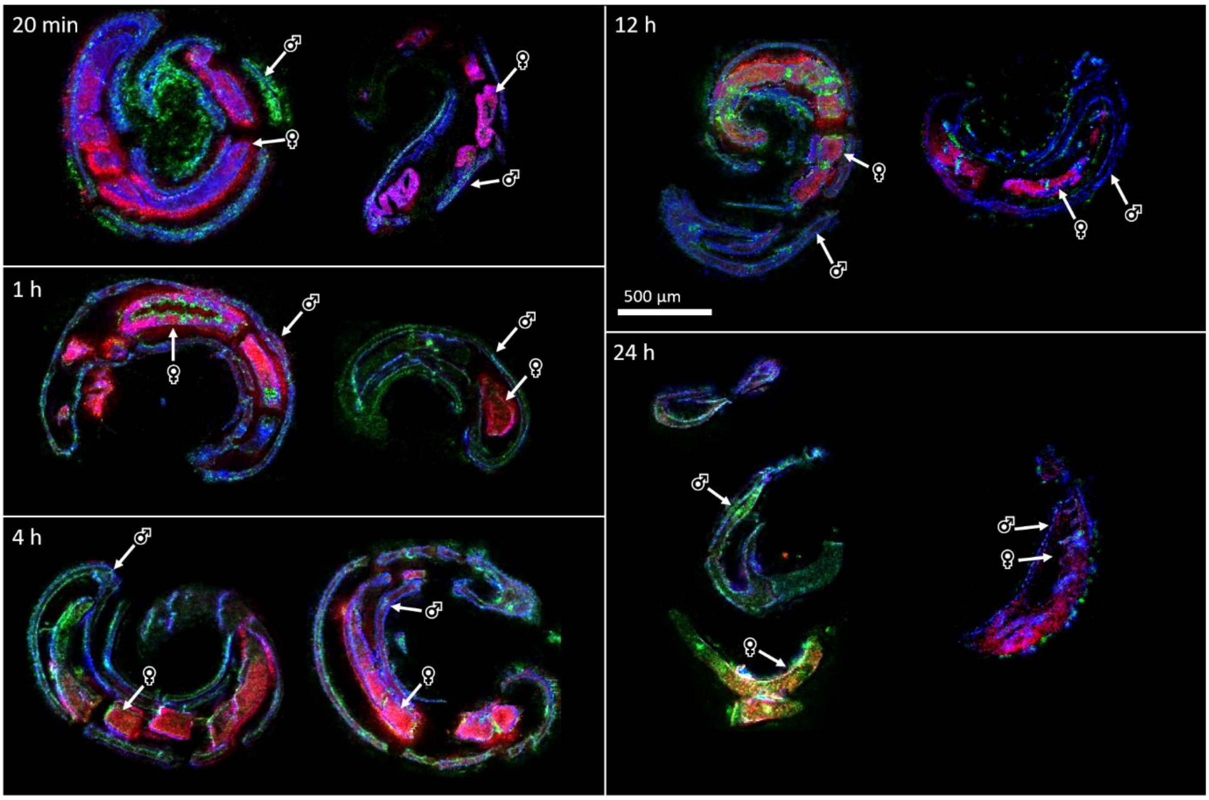

Fig. S11: AP-SMALDI MS images of sections of *S. mansoni* couples after imatinib treatment (20 µmol/l). MS images match with the optical images at the corresponding incubation times in supplementary Fig. S5. Visualized are *m/z* 494.266281 ((20 min), 494.266557 (1 h), 494.266568 (4 h), 494.266670 (12 h), 494.266633 (24 h); green, imatinib; theoretical *m/z*: 494.266284; error: -0.01 ppm (20 min), 0.55 ppm (1 h), 0.57 ppm (4 h), 0.78 ppm (12 h), 0.71 ppm (24 h)), *m/z* 760.584889 ((20 min), 760.584927 (1 h), 760.584919 (4 h), 760.585195 (12 h), 760.585163 (24 h); blue, PC(34:1); theoretical *m/z*: 760.585083; error: -0.26 ppm (20 min), -0.21 ppm (1 h), -0.22 ppm (4 h), 0.15 ppm (12 h), 0.10 ppm (24 h)), and *m/z* 579.534643 ((20 min), 579.534765 (1 h), 579.534745 (4 h), 579.534999 (12 h), 579.535098 (24 h); red, DG(34:0); theoretical *m/z*: 579.534686; error: -0.07 ppm(20 min), 0.14 ppm (1 h), 0.10 ppm (4 h), 0.54 ppm (12 h), 0.71 ppm (24 h)). The experimental values are the average of the samples shown for the respective incubation times.

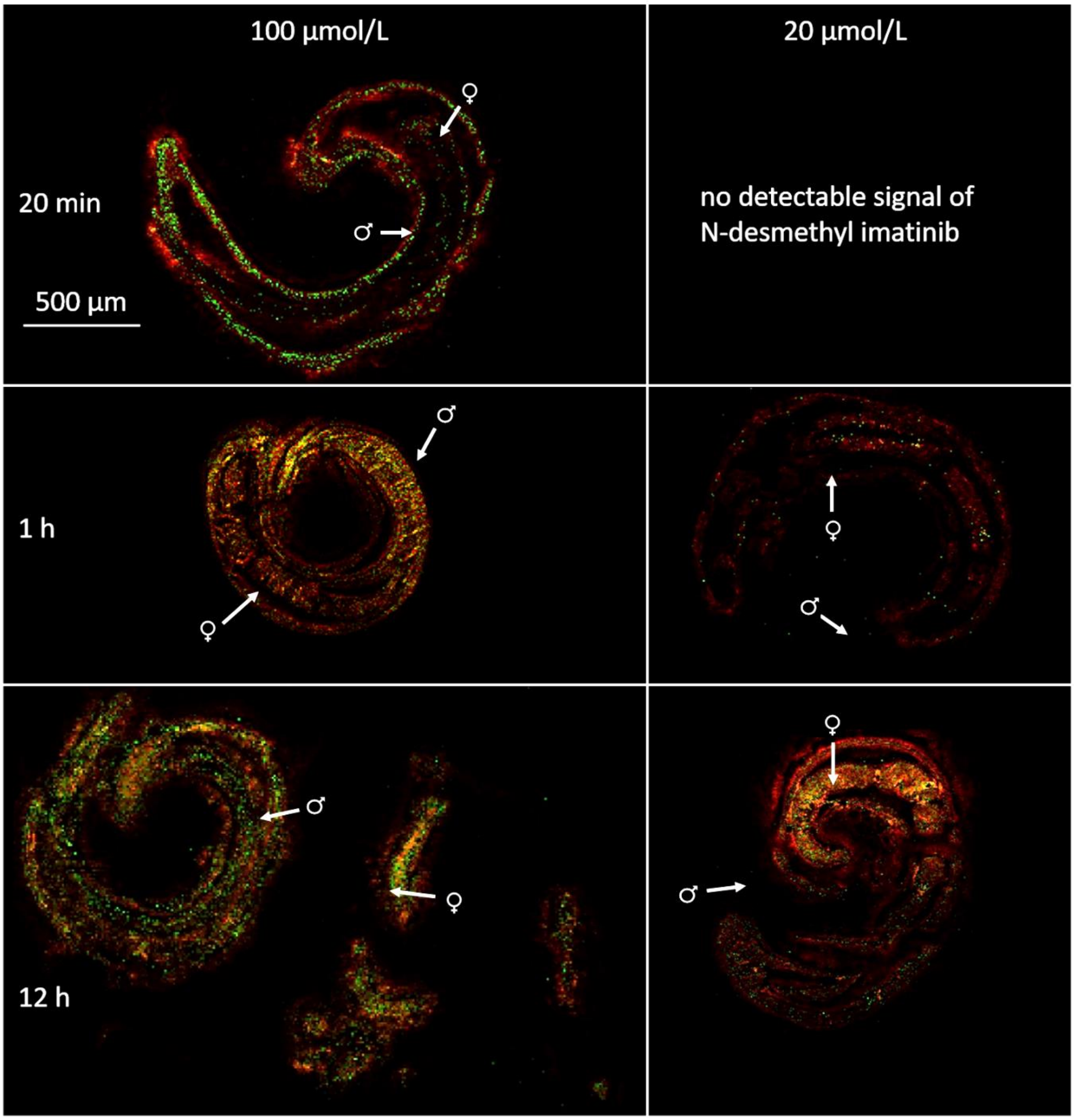

Fig. S12: AP-SMALDI MS images of sections of *S. mansoni* couples after treatment with 100 or 20 µmol/L imatinib. Visualized are *m/z* 494.266308 ((100 µmol/L, 20 min), 494.265444 (100 µmol/L, 1 h), 494.266598 (20 µmol/L, 1 h), 494.26648 (100 µmol/L, 12 h), 494.266546 (20 µmol/L, 12 h); red, imatinib; theoretical *m/z*: 494.266284; error: 0.05 ppm (100 µmol/L, 20 min), -1.70 ppm (100 µmol/L, 1 h), 0.63 ppm (20 µmol/L, 1 h), 0.40 ppm (100 µmol/L, 12 h), 0.53 ppm (20 µmol/L, 12 h)) and *m/z* 480.250609 ((100 µmol/L, 20 min), 480.249815 (100 µmol/L, 1 h), 480.250838 (20 µmol/L, 1 h), 480.250815 (100 µmol/L, 12 h), 480.250879 (20 µmol/L, 12 h); green, N-desmethyl imatinib; theoretical *m/z*: 480.250634; error: -0.05 ppm (100 µmol/L, 20 min), -1.71 ppm (100 µmol/L, 1 h), 0.42 ppm (20 µmol/L, 1 h), 0.38 ppm (100 µmol/L, 12 h), 0.51 ppm (20 µmol/L, 12 h)). Signal intensities of the metabolite were lower for samples with lower imatinib concentrations.

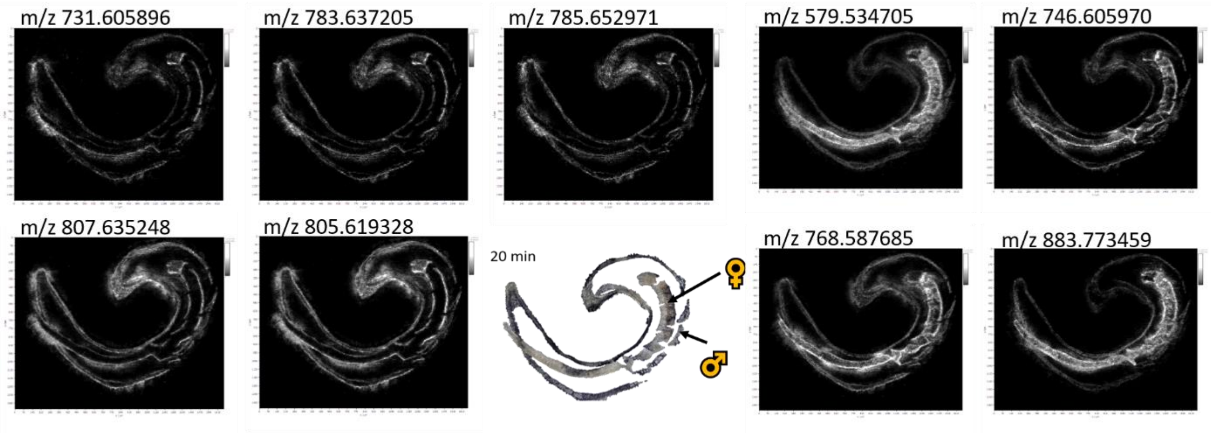

Fig. S13: Sex-specific m/z values that were optically assessed in MSI data and listed in Table 1 in the main manuscript. The picture shows the measurement of a worm, 20 min after incubation with 100 µmol/L imatinib medium. Signals with enhanced signal intensity in the male are depicted on the left side, signals with elevated intensity in the female worm are shown on the right side.

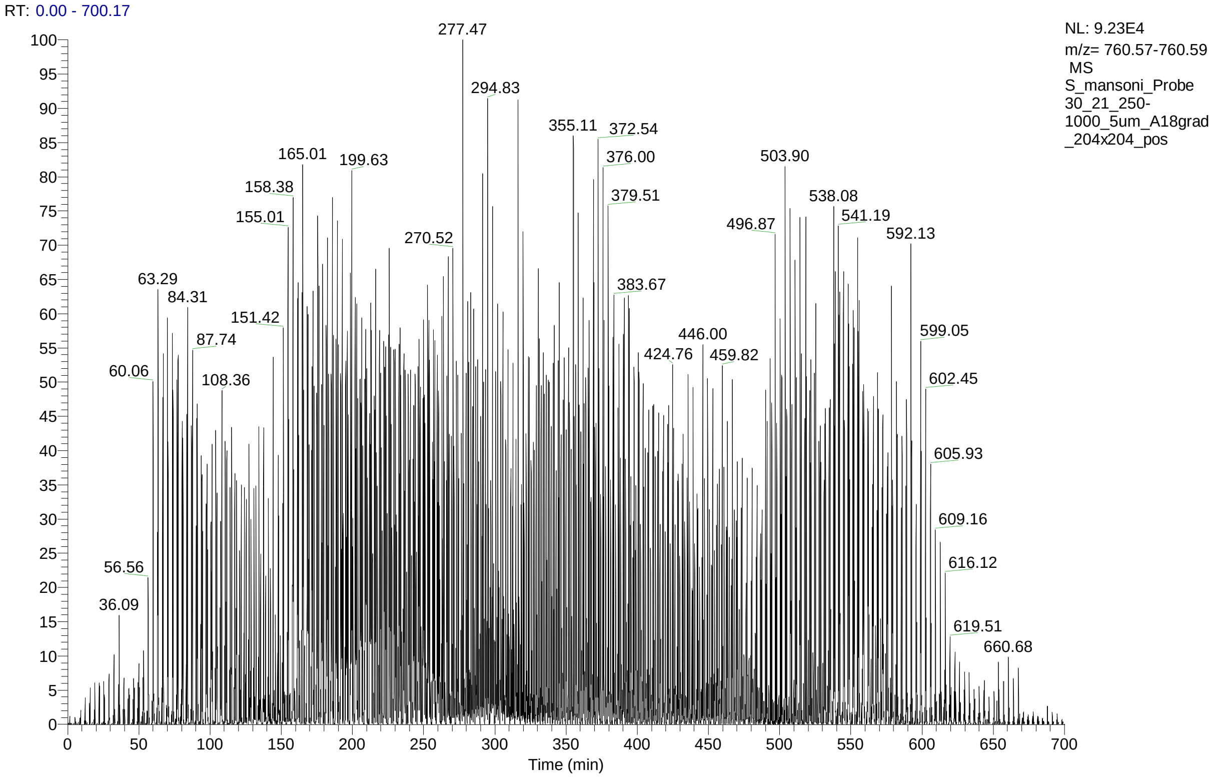

Fig. S14: Signal intensity chromatogram during the whole measurement of the signal with an m/z value of 760.584983 (PC 34:1). Normalized Level (NL) of the base peak in this chromatogram was $9.23*10^4$.

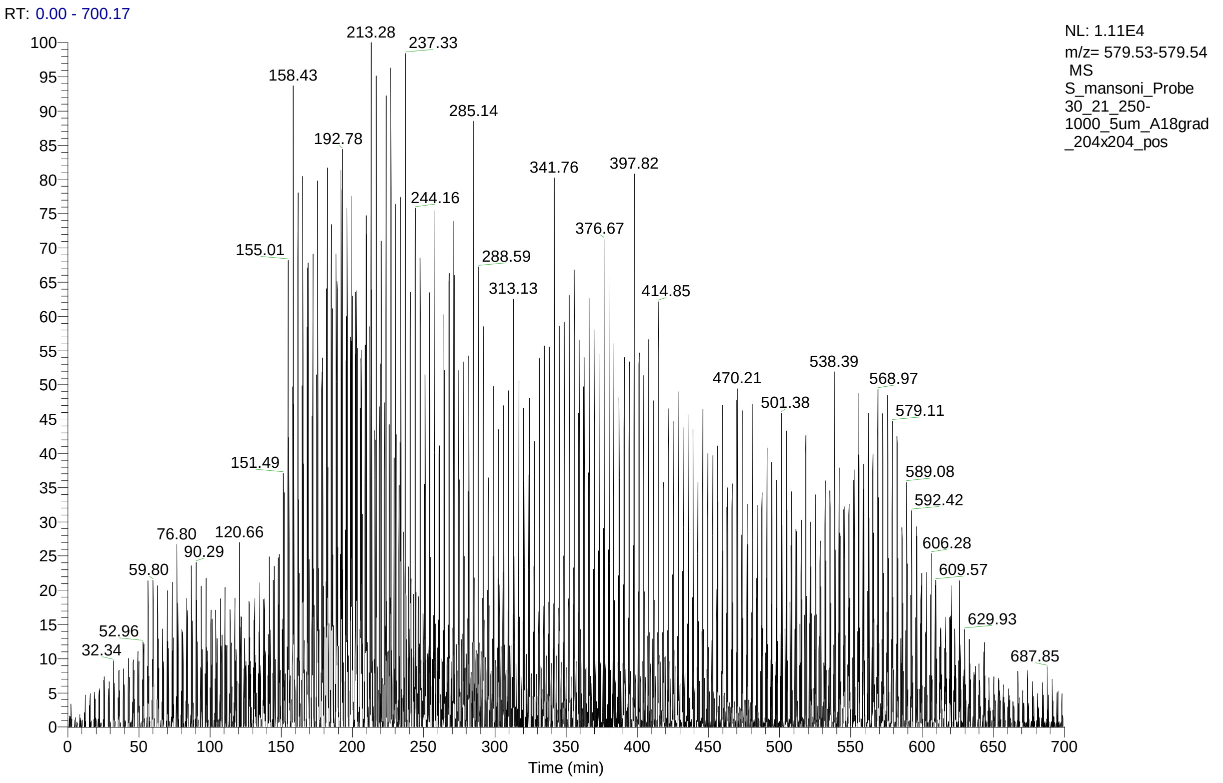

Fig. S15: Signal intensity chromatogram during the whole measurement of the signal with an m/z value of 579.534620 (DG 34:0). Normalized Level (NL) of the base peak in this chromatogram was $1.11*10^4$.

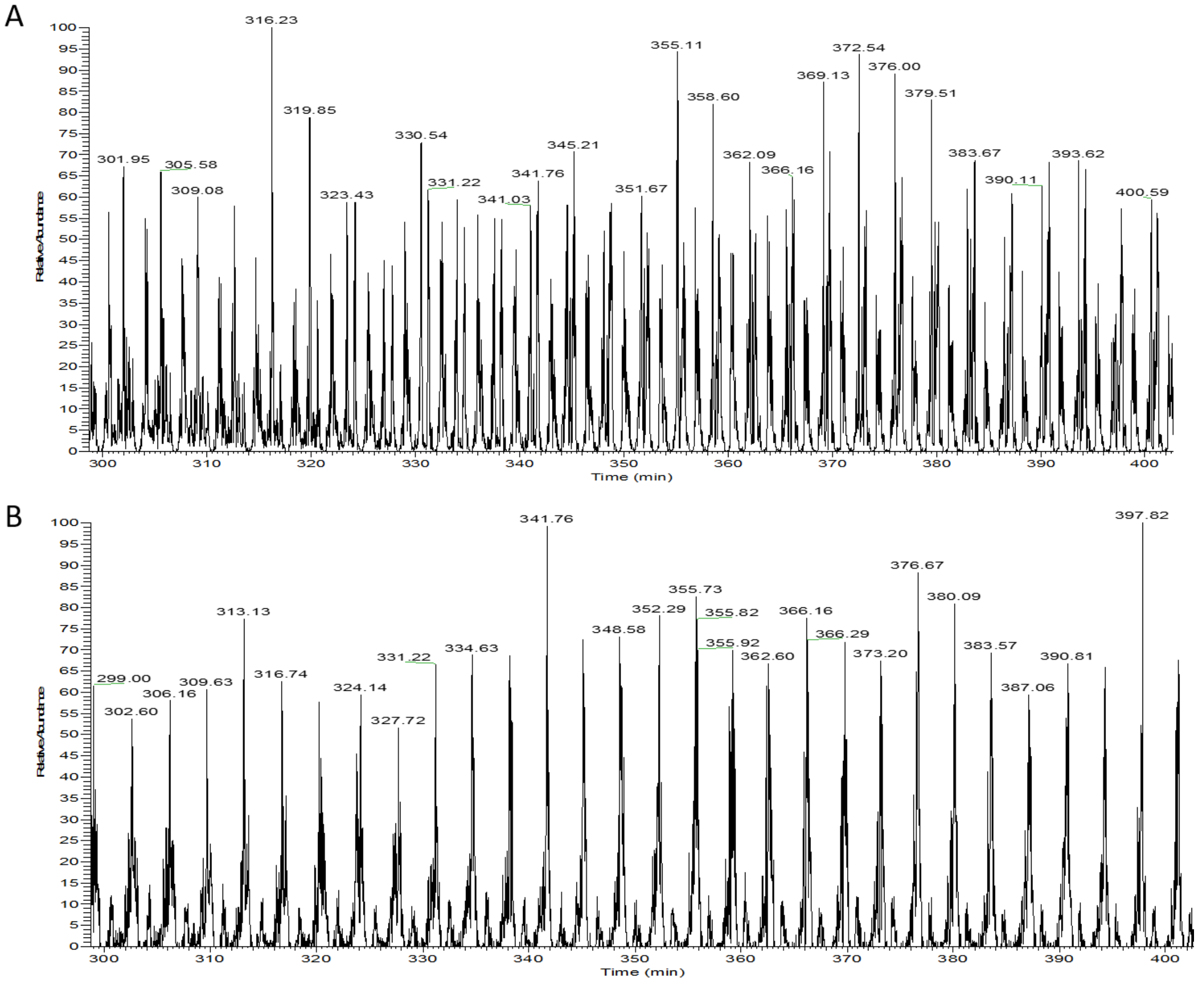

Fig. S16: Excerpt (between 300 and 400 min) of both signal intensity chromatograms of supplementary figures S14 and S15. A) showing the signal intensities of the signal with an m/z value of 760.584983 (PC 34:1) and B) showing the signal intensities of the signal with an m/z value of 579.534620 (DG 34:0).
